# P‐tau235: a novel biomarker for staging preclinical Alzheimer’s disease

**DOI:** 10.15252/emmm.202115098

**Published:** 2021-11-02

**Authors:** Juan Lantero‐Rodriguez, Anniina Snellman, Andrea L Benedet, Marta Milà‐Alomà, Elena Camporesi, Laia Montoliu‐Gaya, Nicholas J Ashton, Agathe Vrillon, Thomas K Karikari, Juan Domingo Gispert, Gemma Salvadó, Mahnaz Shekari, Christina E Toomey, Tammaryn L Lashley, Henrik Zetterberg, Marc Suárez‐Calvet, Gunnar Brinkmalm, Pedro Rosa Neto, Kaj Blennow

**Affiliations:** ^1^ Department of Psychiatry and Neurochemistry Institute of Neuroscience & Physiology Sahlgrenska Academy at the University of Gothenburg Mölndal Sweden; ^2^ Turku PET Centre University of Turku Turku Finland; ^3^ Translational Neuroimaging Laboratory McGill Centre for Studies in Aging McGill University Montreal QC Canada; ^4^ Barcelonaβeta Brain Research Center (BBRC) Pasqual Maragall Foundation Barcelona Spain; ^5^ Hospital del Mar Medical Research Institute (IMIM) Barcelona Spain; ^6^ Centro de Investigación Biomédica en Red de Fragilidad y Envejecimiento Saludable (CIBERFES) Madrid Spain; ^7^ Universitat Pompeu Fabra Barcelona Spain; ^8^ Wallenberg Centre for Molecular and Translational Medicine University of Gothenburg Gothenburg Sweden; ^9^ Department of Old Age Psychiatry Maurice Wohl Clinical Neuroscience Institute King’s College London London UK; ^10^ NIHR Biomedical Research Centre for Mental Health & Biomedical Research Unit for Dementia at South London & Maudsley NHS Foundation London UK; ^11^ Cognitive Neurology Center GHU Nord APHP Hospital Lariboisière Fernand Widal Université de Paris Paris France; ^12^ Inserm UMR S11‐44 Therapeutic Optimization in Neuropsychopharmacology Université de Paris Paris France; ^13^ Department of Psychiatry University of Pittsburgh Pittsburgh PA USA; ^14^ Centro de Investigación Biomédica en Red Bioingeniería Biomateriales y Nanomedicina (CIBER‐BBN) Madrid Spain; ^15^ The Queen Square Brain Bank for Neurological Disorders Department of Clinical and Movement Neurosciences UCL Institute of Neurology London UK; ^16^ Department of Neurodegenerative Disease UCL Institute of Neurology University College London London UK; ^17^ Clinical Neurochemistry Laboratory Sahlgrenska University Hospital Mölndal Sweden; ^18^ UK Dementia Research Institute at UCL London UK; ^19^ Hong Kong Center for Neurodegenerative Diseases Hong Kong China; ^20^ Servei de Neurologia Hospital del Mar Barcelona Spain; ^21^ Montreal Neurological Institute Montreal QC Canada; ^22^ Department of Neurology and Neurosurgery McGill University Montreal QC Canada

**Keywords:** Alzheimer’s disease, biomarkers, CSF, p‐tau235, tau, Biomarkers, Neuroscience

## Abstract

Alzheimer’s disease (AD) is characterised by a long preclinical phase. Although phosphorylated tau (p‐tau) species such as p‐tau217 and p‐tau231 provide accurate detection of early pathological changes, other biomarkers capable of staging disease progression during preclinical AD are still needed. Combining exploratory and targeted mass spectrometry methods in neuropathologically confirmed brain tissue, we observed that p‐tau235 is a prominent feature of AD pathology. In addition, p‐tau235 seemed to be preceded by p‐tau231, in what appeared to be a sequential phosphorylation event. To exploit its biomarker potential in cerebrospinal fluid (CSF), we developed and validated a new p‐tau235 Simoa assay. Using three clinical cohorts, we demonstrated that (i) CSF p‐235 increases early in AD *continuum*, and (ii) changes in CSF p‐tau235 and p‐tau231 levels during preclinical AD are consistent with the sequential phosphorylation evidence in AD brain. In conclusion, CSF p‐tau235 appears to be not only a highly specific biomarker of AD but also a promising staging biomarker for the preclinical phase. Thus, it could prove useful tracking disease progression and help enriching clinical trial recruitment.

## Graphical abstract

A combination of mass spectrometry analysis of brain tissue and ultrasensitive immunoassays in three independent CSF cohorts identifies p‐tau235 as a novel biomarker for Alzheimer's disease (AD) – from preclinical stages to dementia.

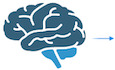

**The paper explained**

**Problem**

Despite the success in the development of fluid biomarkers to detect Alzheimer’s disease (AD) pathology, there remains a need for biomarkers to characterise the long preclinical stage of the disease. It is becoming clear in the AD field that a detailed understanding of the preclinical stage is essential to develop primary and secondary strategies to prevent this fatal disease. Currently, highly accurate biomarkers are available to detect preclinical AD (e.g., elevated amyloid‐β pathology but no cognitive deficit). However, these biomarkers are not optimal in determining if an individual is at the beginning of this 15–20 year preclinical period or imminently progressing to cognitive impairment.

**Results**

We first examined neuropathologically confirmed AD and control brain tissue and employed a phosphoproteomic mass spectrometry method which highlighted the prominence of p‐tau235 in AD pathology. Our findings strongly suggest that threonine 231 and serine 235 undergo sequential phosphorylation as a result of AD pathology, and would pose a great potential to track disease progression. Using an in‐house developed Simoa assay specifically targeting CSF p‐tau235, we measured this p‐tau species in three independent CSF cohorts, demonstrating that CSF p‐tau235 is a highly specific novel biomarker to detect AD. Moreover, we show the predominance of the double‐positive p‐tau231/235 in individuals classified as late‐stage preclinical AD, while single positivity for CSF p‐tau231 was shown to be a more defining feature of early preclinical disease.

**Impact**

These results have great potential in the design of observational or interventional studies at the preclinical stage of the disease: a study aiming to target the earliest stage of preclinical AD would include single positive p‐tau231 participants, whereas a study in asymptomatic individuals but closer to symptom onset would include double‐positive p‐tau231/235 participants. In trials evaluating disease‐modifying drugs, which are increasingly focusing on the preclinical stage of AD, p‐tau235 could be used as an inclusion or exclusion biomarker depending on the preclinical stage being targeted.

## Introduction

The 2018 world Alzheimer’s disease (AD) report estimated that 50 million people worldwide suffered from dementia and that the number of cases would triple by 2050, which is paralleled by an increase in the medical costs estimated as 2 trillion US$ by 2030. AD emerges as the most prevalent cause of dementia, accounting for 50–60% of all cases (Patterson, 2018). AD is neuropathologically characterised by the aberrant aggregation of amyloid‐β (Aβ) into extracellular plaques as well as hyperphosphorylated tau protein into intraneuronal neurofibrillary tangles (NFTs) and dystrophic neurites surrounding the plaques, which together represent the hallmarks of the disease (Braak & Braak, 1991; Thal *et al*, 2002). To this day, post‐mortem examination confirming the presence of both NFTs and Aβ plaques is required for definitive diagnosis of AD (Blennow *et al*, 2006; Jack *et al*, 2013). On the other hand, the clinical diagnosis of AD is increasingly supported by imaging and cerebrospinal fluid (CSF) biomarkers. Imaging biomarkers include positron emission tomography (PET) using radiotracers capable of specifically binding tau aggregates (tau PET) (Scholl *et al*, 2019) and fibrillary Aβ deposits (Aβ PET) (Ashton *et al*, 2018), whereas the ‘core’ CSF biomarkers include Aβ_1–42_ (or Aβ_1–42/40_ ratio), phosphorylated tau at threonine 181 (p‐tau181) and total tau (t‐tau) (Molinuevo *et al*, 2018). Decreased levels of CSF Aβ_1–42_ reflect Aβ burden in the brain as a result of its accumulation into plaques, whereas p‐tau181 and t‐tau have been suggested to reflect increased tau phosphorylation and general tau secretion from Aβ‐affected neurons (both appear predictive of AD‐type tangle pathology and neurodegeneration, respectively) (Molinuevo *et al*, 2018).

During recent years, novel mass spectrometry (MS) and antibody‐based methods have greatly contributed to our knowledge regarding the time course of different p‐tau species in fluids, helping to further understand their potential as biomarkers of AD pathology during disease progression (Barthelemy *et al*, 2019, 2020; Karikari *et al*, 2020; Lantero Rodriguez *et al*, 2020; Palmqvist *et al*, 2020; Suarez‐Calvet *et al*, 2020; preprint: Ashton *et al*, 2021a; Ashton, *et al*, 2021b). In this context, a growing body of evidence challenges the traditional view of p‐tau as a biomarker of NFT pathology in the brain. Firstly, only modest correlations have been observed between CSF phosphorylated tau (p‐tau) and *in vivo* measurements of NFTs by tau PET, and secondly, CSF p‐tau appears to increase before tangle formation defined by tau PET positivity (Mattsson *et al*, 2017; Scholl *et al*, 2019). In addition, a recent study including only cognitively unimpaired (CU) individuals demonstrated that the abnormal increase in different p‐tau species such as p‐tau217 or p‐tau231 occurred early within preclinical AD, when only minor changes in Aβ pathology can be detected in CSF (Suarez‐Calvet *et al*, 2020). Thus, it has been hypothesised that neuronal exposure to Aβ during early phases of preclinical AD might be reflected on the gradual appearance of different p‐tau species in CSF (preprint: Ashton *et al*, 2021a; Ashton *et al*, 2021b). Thus, these biomarkers could reflect tau phosphorylation and release in response to emerging or incipient Aβ aggregation, rather than tangle pathology.

The recent availability of different p‐tau biomarkers such as p‐tau217 and p‐tau231 capable of detecting very early AD pathological processes (Suarez‐Calvet *et al*, 2020) has highlighted the additional need for a staging biomarker for the long asymptomatic phase of the disease. In search of such biomarkers, the phosphorylation pattern of insoluble tau in AD brain may provide targets of interest. MS analysis of paired helical filaments (PHFs) extracted from AD brains indicates that phosphorylation may occur in a temporarily ordered manner, affecting different clusters of threonine and serine amino acids along the tau sequence (Hanger *et al*, 2007). In other words, it appears that the addition of phosphoryl groups to certain amino acids within these clusters primes the subsequent phosphorylation at nearby threonine and serine residues, thus generating mutually exclusive combinations of phosphorylation. Interestingly, three of the reported clusters include phosphorylations of great interest from the fluid biomarker perspective, such as p‐tau181, p‐tau217 or p‐tau231, which not only are readily measurable in CSF and blood but also have been shown to increase very early in the disease course, in preclinical AD (Suarez‐Calvet *et al*, 2020). Most importantly, these three phosphorylations seemed to be primordial events on each cluster. Thus, if the sequential phosphorylation‐affecting PHFs in AD brain translates into fluid, then the phosphorylations at nearby residues of p‐tau181, p‐tau217 or p‐tau231 pose great potential to track disease progression.

In this study, we used MS to investigate soluble fractions from brain homogenates to identify novel p‐tau species with biomarker potential. Based on our findings in the brain, p‐tau235 was found to be a very promising target, which belongs to the serine and threonine cluster found between amino acids 230 and 240, together with p‐tau231. Considering this, we developed an ultrasensitive in‐house immunoassay to specifically quantify p‐tau235 in CSF and measured this novel biomarker in three independent cohorts including participants at different stages of the AD *continuum*, with a special focus on preclinical AD. Furthermore, the performance of CSF p‐tau235 was compared with other novel CSF p‐tau biomarkers, namely, CSF p‐tau217 and p‐tau231.

## Results

### Immunoprecipitation–mass spectrometry analysis of p‐tau species in brain tissue extracts

**Figure 1:**
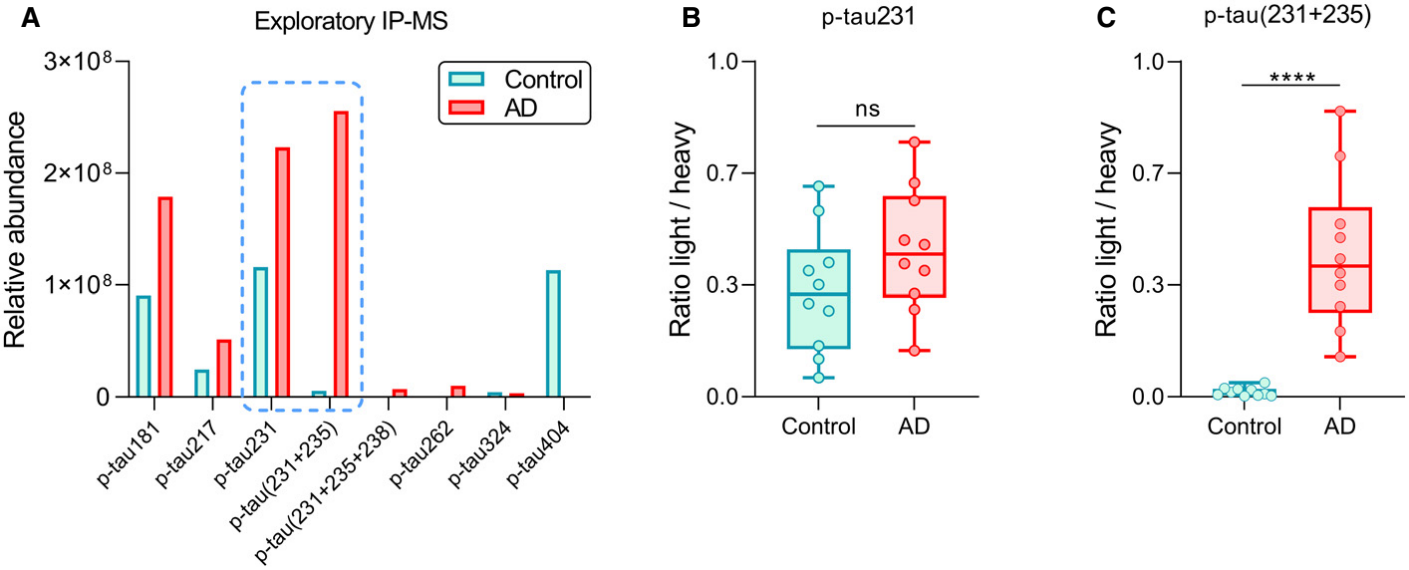
Exploratory and targeted IP‐MS in brain Bar chart showing the relative abundance (signal intensity) of the phosphorylated tau species identified using an exploratory IP‐MS approach using HT7 antibody on TBS‐soluble control and Alzheimer’s disease (AD) pools. Blue dashed line indicates the two phosphorylated tau peptides that underwent targeted IP‐MS: mono‐phosphorylated p‐tau231 and double‐phosphorylated p‐tau(231+235).Box‐and‐whisker plot showing quantification of mono‐phosphorylated p‐tau231 in control (*n* = 10) and AD (*n* = 10) TBS‐soluble fractions using a targeted IP‐MS method (*P* > 0.05, non‐significant, ns, Mann–Whitney *U* test).Box‐and‐whisker plot showing the quantification of double‐phosphorylated p‐tau(231+235) in control (*n* = 10) and AD (*n* = 10) TBS‐soluble fractions using a targeted IP‐MS method. Data information: Box‐and‐whisker plots show the median and the 25^th^ and 75^th^ percentiles. Group differences determined using Mann‐Whitney *U* test (****P < 0.0001). All samples were run in singlicates.

We first investigated the phosphorylation pattern of tau in Tris‐buffered saline (TBS) soluble fraction of human cortical brain homogenates. We used the TBS‐soluble material because the CSF composition partially reflects this fraction (Buerger *et al*, 2006; Barthelemy *et al*, 2019). An exploratory immunoprecipitation–mass spectrometry (IP‐MS) experiment using HT7 antibody (epitope aa159–163, reacting with all tau isoforms and both phosphorylated and non‐phosphorylated tau species) in AD and control pools showed that mono‐phosphorylated tryptic peptides containing p‐tau181, p‐tau217 and p‐tau231 were among the four most relevant p‐tau species in terms of relative abundance (signal intensity) (Fig 1A). Moreover, these peptides were more prominent in AD than control TBS pool, which resembles CSF findings. Interestingly, the tau species with the highest relative abundance was the double‐phosphorylated tau tryptic peptide containing p‐tau231 and p‐tau235 (referred to as p‐tau(231+235)), which was found almost exclusively in AD TBS‐soluble pool (Fig 1A). In contrast, mono‐phosphorylated p‐tau235 was not detected. Subsequent results from a targeted IP‐MS experiment with HT7 antibody in each individual TBS fraction showed that mono‐phosphorylated p‐tau231 was not significantly increased in AD (*P* = 0.17), although it displayed a moderate increasing trend (Fig 1B). On the contrary, double‐phosphorylated p‐tau(231+235) was highly increased in AD cases when compared with controls (*P* < 0.0001) (Fig 1C). Additionally, detailed examination of MS/MS spectra generated during the exploratory and targeted IP‐MS did not reveal any other p‐tau235‐containing species besides the double‐phosphorylated p‐tau(231+235) (Appendix Fig S2). Thus, our results showed that the double‐phosphorylated tau tryptic peptide p‐tau(231+235), but not the mono‐phosphorylated p‐tau231, is highly and specifically increased in brains of patients with AD at Braak stages V/VI.

### CSF p‐tau235 in the discovery cohort

**Figure 2:**
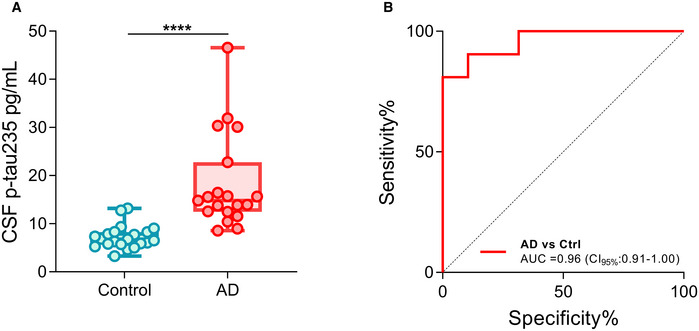
CSF p‐tau235 performance in the discovery cohort Box‐and‐whiskers plot showing the levels of CSF p‐tau235 in neurological controls (*n* = 21) and biologically defined Alzheimer’s disease (AD) cases (*n* = 19).ROC analysis indicating the diagnostic accuracy of CSF p‐tau235 when discriminating AD from control. Data information: Box‐and‐whisker plots show the median and the 25^th^ and 75^th^ percentiles. Group differences determined using Mann–Whitney *U* test (****P < 0.0001). All samples were run in singlicates.

We next investigated p‐tau235 in the discovery cohort using our in‐house Simoa assay. The cohort comprised AD patients with a typical AD CSF profile and neurological controls with minor neurological or psychiatric symptoms. CSF p‐tau235 was significantly increased in AD when compared with controls (*P* < 0.0001) (Fig 2A). CSF p‐tau235 had a high diagnostic performance to identify AD cases (AUC = 0.96, CI_95%_ = 0.91–1.00) (Fig 2B). In addition, CSF p‐tau235 strongly correlated with all three core CSF biomarkers for AD, specifically a negative correlation with Aβ_1–42_ (*r*_S_ = −0.77, *P* < 0.0001) and positive correlations with t‐tau (*r*_S_ = 0.85, *P* < 0.0001) and p‐tau181 (*r*_S_ = 0.78, *P* < 0.0001) (Appendix Table S4).

### CSF p‐tau235 in the whole AD *continuum*: the TRIAD cohort

**Figure 3:**
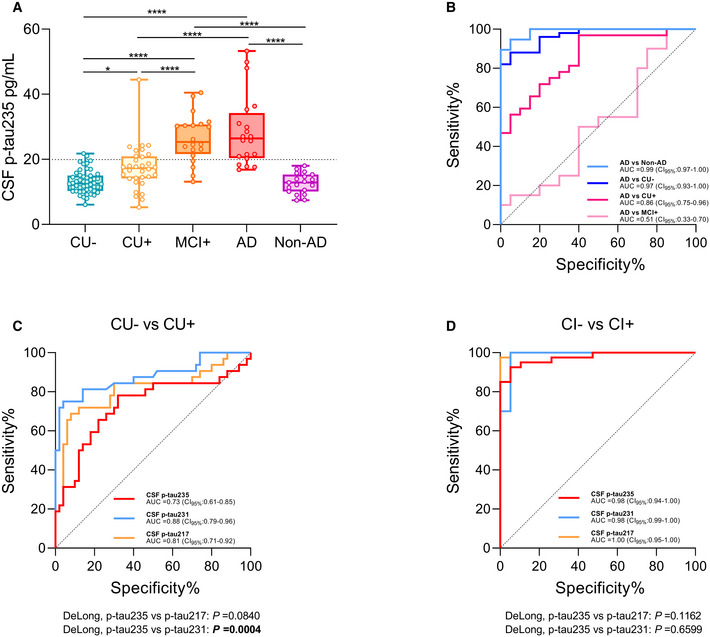
CSF p‐tau235 assay across Alzheimer’s disease *continuum* (TRIAD cohort) Box‐and‐whiskers plot showing CSF p‐tau235 concentrations in the different groups: amyloid‐negative cognitively unimpaired (CU−) participants (*n* = 50), participants across Alzheimer’s disease (AD) *continuum* (amyloid‐positive cognitively unimpaired (CU+, *n* = 32); amyloid‐positive mild cognitive impairment (MCI+, *n* = 20); AD (*n* = 20) and non‐AD cases (*n* = 19)). CSF p‐tau235 is highly specific for AD pathology. Cut‐off value for CSF p‐tau235 positivity is displayed with black dashed line (19.92 pg/ml).ROC analysis showing the high diagnostic accuracy of CSF p‐tau235 discriminating AD from CU−, CU+, MCI+ and non‐AD groups.ROC analysis comparing AUC values of CSF p‐tau235, p‐tau231 and p‐tau217 discriminating CU+ from CU−. CSF p‐tau235 discriminating accuracy was statistically lower than that of CSF p‐tau231, but not CSF p‐tau217.ROC analysis comparing AUC values of CSF p‐tau235, p‐tau231 and p‐tau217 discriminating amyloid‐negative cognitively impaired (CI−, non‐AD) from amyloid positive cognitively impaired (CI+, MCI+ and AD). CSF p‐tau235 performance was statistically similar to that of CSF p‐tau217 and p‐tau231. Data information: Box‐and‐whisker plots show the median and the 25^th^ and 75^th^ percentiles. *P*‐values determined using one‐way ANOVA adjusted by age and sex followed by Bonferroni‐corrected post‐hoc comparison (*P < 0.05, ****P < 0.0001). CSF p‐tau235 cut‐off was determined as mean ± 2 SD of the A−T− group in ALFA+ cohort (19.92 pg/ml). ROC analysis (B–D) indicating the diagnostic accuracy of the studied biomarkers as AUC values. The DeLong test (C, D) was used to determine statistical differences between biomarker performances (*P* < 0.05 is indicated in bold). All samples were run in singlicates.

The TRIAD cohort is a cross‐sectional study with participants ranging across the AD continuum, with detailed clinical and cognitive assessment and a wide range of biomarker data (both fluid and imaging). In the TRIAD cohort, CSF p‐tau235 was significantly increased in AD when compared with non‐AD (*P* < 0.0001), cognitively unimpaired Aβ pathology negative (CU−, *P* < 0.0001) and cognitively unimpaired Aβ pathology positive (CU+, *P* < 0.0001). Similarly, CSF p‐tau235 was significantly increased in mild cognitive impairment due to AD (Aβ pathology positive) (MCI+) when compared with non‐AD (*P* < 0.0001), CU− (*P* < 0.0001) and CU+ (*P* < 0.0001) (Fig 3A). We also observed a subtle, although significant, increase in CSF p‐tau235 in CU+, when comparing the asymptomatic groups CU− and CU+ (*P* = 0.040), which indicates that CSF p‐tau235 emerges very early in the AD *continuum*, specifically during preclinical stages (Fig 3A). CSF p‐tau235 exhibited high accuracy discriminating MCI+ and AD from non‐AD (AUC_AD vs Non‐AD_ = 0.99, CI_95%_ = 0.97–1.00; AUC_MCI vs Non‐AD_ = 0.96, CI_95%_ = 0.90–1.00), CU− (AUC_AD vs CU−_ = 0.97, CI_95%_ = 0.93–1.00; AUC_MCI+ vs CU−_ = 0.96, CI_95%_ = 0.90–1.00) and CU+ (AUC_AD vs CU+_ = 0.86, CI_95%_ = 0.75–0.96; AUC_MCI+ vs CU+_ = 0.84, CI_95%_ = 0.72–0.96) (Fig 3B).

We then compared CSF p‐tau235 performance with CSF p‐tau217 and p‐tau231. The accuracy of CSF p‐tau235 distinguishing CU+ from CU‐ (AUC_235_ = 0.73, CI_95%_ = 0.61–0.85) was significantly lower than that of CSF p‐tau231 (AUC_231_ = 0.88, CI_95%_ = 0.79–0.96; DeLong_231–235_ *P* = 0.0004), but not significantly different from that of CSF p‐tau217 (AUC_217_ = 0.81, CI_95%_ = 0.71–0.92; DeLong_217–235_ *P* = 0.084) (Fig 3C). In contrast, all p‐tau biomarkers had a very similar accuracy when distinguishing cognitively impaired Aβ pathology negative or CI− (non‐AD) from cognitively impaired Aβ pathology positive or CI+ (MCI+ and AD) cases (AUC_235_ = 0.98, CI_95%_ = 0.94–1.00; AUC_217_ = 1.00, CI_95%_ = 0.99–1.00; AUC_231_ = 0.98, CI_95%_ = 0.95–1.00; DeLong_217–235_ *P* = 0.12, DeLong_231–235_ *P* = 0.66) (Fig 3D). In addition, CSF p‐tau235 was highly correlated with both CSF p‐tau217 (*r*_S_ = 0.87, *P* < 0.0001,) and p‐tau231 (*r*_S_ = 0.89, *P* < 0.0001) (Appendix Table S4). We also investigated the association of CSF p‐tau235 with both Aβ and tau PET. CSF p‐tau235 was significantly associated with Aβ PET (*r*_S_ = 0.66 *P* < 0.0001) and tau PET (*r*_S_ = 0.66, *P* < 0.0001) in the whole cohort. Within the respective groups of PET‐positive cases, CSF p‐tau235 correlated slightly better with the tau PET (*r*_S_ = 0.65, *P* < 0.0001) than with the Aβ PET (*r*_S_ = 0.58, *P* < 0.0001) (Appendix Fig S3). We further explored the association of p‐tau235 with *in vivo* tau pathology by examining the levels of this biomarker across the different Braak stages. A subtle, albeit non‐significant, increase in p‐tau235 concentrations was observed between Braak 0 and I/II (*P* = 0.095), followed by a very prominent increase between Braak I/II and III/IV (*P* < 0.0026). No significant differences could be found between Braak III/IV and V/VI (*P* = 1.0), although CSF p‐tau235 appeared to display an increasing trend (Appendix Fig S4).

### CSF p‐tau235 in preclinical AD: the ALFA+ cohort

**Figure 4:**
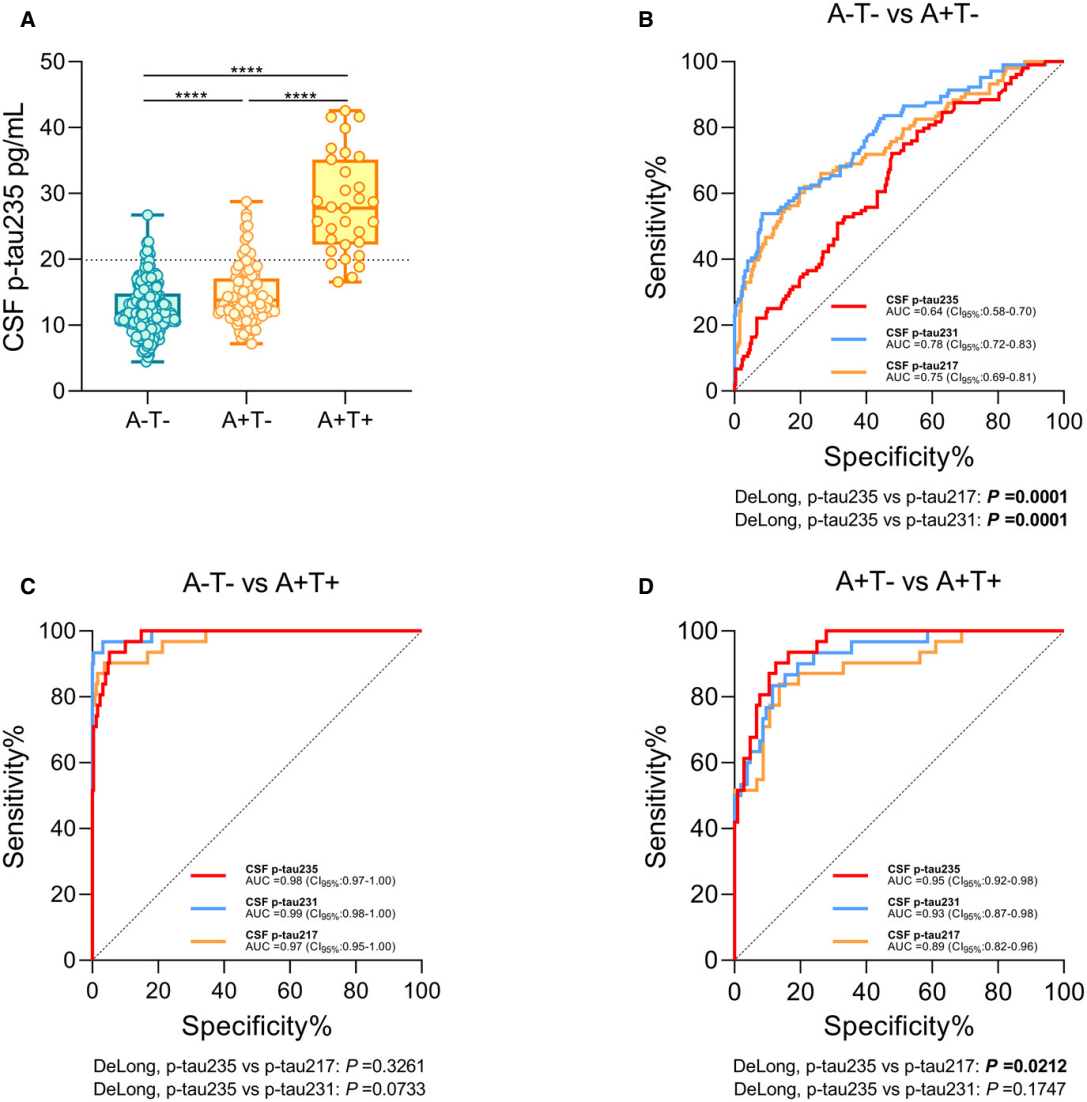
CSF p‐tau235 assay across preclinical Alzheimer’s disease (ALFA+ cohort) Box‐and‐whiskers plot showing CSF p‐tau235 concentrations in A−T− participants and across preclinical Alzheimer’s disease (AD, dichotomised as A+T− and A+T+). CSF p‐tau235 was increased already in A+T−, which represents the early cases within preclinical AD. A prominent increase was observed from A+T− to A+T+, the latter representing late preclinical AD cases. Cut‐off value for CSF p‐tau235 positivity is displayed with black dashed line (19.92 pg/ml).ROC analysis comparing AUC values of CSF p‐tau235, p‐tau231 and p‐tau217 discriminating A−T− from A+T−. Both CSF p‐tau217 and p‐tau231 showed a statistically higher accuracy discriminating A−T− from A+T− than CSF p‐tau235.ROC analysis comparing AUC values of CSF p‐tau235, p‐tau231 and p‐tau217 discriminating A−T− from A+T+. All three assays had statistically the same accuracy when distinguishing A−T− from A+T+.ROC analysis comparing AUC values of CSF p‐tau235, p‐tau231 and p‐tau217 discriminating A+T− from A+T+. CSF p‐tau235 demonstrated statistically superior diagnostic accuracy compared with CSF p‐tau217 discriminating the two preclinical groups (A+T− and A+T+). In this scenario, no differences in diagnostic performance between CSF p‐tau235 and p‐tau231 were observed. Data information: Box‐and‐whisker plots show the median and the 25^th^ and 75^th^ percentiles. *P*‐values were determined using one‐way ANOVA adjusted by age and sex, followed by Bonferroni‐corrected post‐hoc comparison (****P < 0.0001). The CSF p‐tau235 cut‐off was determined as the mean ± 2 SD of the A−T− group in ALFA+ cohort (19.92 pg/ml). ROC analysis (B–D) indicating biomarker diagnostic accuracy as AUC values. The DeLong test (B–D) was used to determine statistical differences between biomarker performances (*P* < 0.05 is indicated in bold). All samples were run in singlicates.

The ALFA+ study is a cohort exclusively composed of CU participants and has the specific goal of investigating the preclinical stage of AD. Participants in ALFA+ are classified based on the presence (+/−) of Aβ (A) and tau pathology (T) in CSF into three groups: A−T−, A+T− and A+T+. Thus, we leverage the unique characteristics of the ALFA+ cohort to study CSF p‐tau235 in preclinical AD. We found that CSF p‐tau235 was mildly, but significantly, increased in the A + T− when compared with the A−T− group (Cohen’s d (d)_235_ = 0.49, *P* < 0.0001) (Fig 4A), further supporting that this CSF biomarker increases in the preclinical stage of AD, when the CSF Aβ_1–42/40_ ratio is decreased, but CSF p‐tau181 is still not changed. However, the effect size of the increase in CSF p‐235 between A−T− and A+T− was lower than that of CSF p‐tau231 and p‐tau217 (*d*_231_ = 1.12, *P*_231_ < 0.0001; *d*_217_ = 0.92, *P*_217_ < 0.0001). In contrast, CSF p‐tau235 displayed a prominent increase between the A+T− and A+T+ groups (*d*_235_ = 2.18, *P* < 0.0001) (Fig 4A), whereas CSF p‐tau231 and p‐tau217 also significantly increased (*d*_231_ = 2.00, *P*_231_ < 0.0001; *d*_217_ = 1.66, *P*_217_ < 0.0001). We performed ROC analyses to determine the accuracy of CSF p‐tau235 to discriminate between the different AT groups and compared it with that of CSF p‐tau217 and p‐tau231 (Fig 4B–D). Both CSF p‐tau217 and p‐tau231 showed a superior accuracy discriminating A+T− from A−T− compared with CSF p‐tau235 (AUC_235_ = 0.64, CI_95%_ = 0.58–0.70; AUC_217_ = 0.75, CI_95%_ = 0.69–0.81; AUC_231_ = 0.78, CI_95%_ = 0.72–0.83; *DeLong*_217–235_ P = 0.0001, *DeLong*_231–235_ *P* = 0.0001) (Fig 4B). Yet, CSF p‐tau235 had a similar accuracy to CSF p‐tau217 and p‐tau231 when distinguishing A+T+ from A‐T‐ (AUC_235_ = 0.98, CI_95%_ = 0.97–1.00; AUC_217_ = 0.97, CI_95%_ = 0.95–1.00; AUC_231_ = 0.99, CI_95%_ = 0.98–1.00; *DeLong*_217–235_ *P* = 0.33, *DeLong*_231–235_ *P* = 0.073) (Fig 4C). We also determined the accuracy of CSF p‐tau biomarkers to discriminate between the two preclinical AD groups, namely, A+T− and A+T+. In this scenario, CSF p‐tau235 outperformed p‐tau217 (AUC_235_ = 0.95, CI_95%_ = 0.92–0.98; AUC_217_ = 0.89, CI_95%_ = 0.82–0.96; *DeLong*_217–235_ *P* = 0.021*)* but was not better than CSF p‐tau231 (AUC_231_ = 0.93, CI_95%_ = 0.87–0.98; *DeLong*_231–235_ *P* = 0.18) (Fig 4D).

**Figure 5:**
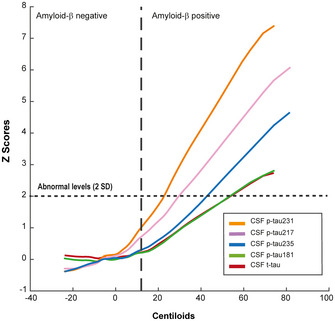
CSF biomarker trajectories in preclinical AD Trajectories of the different tau biomarkers in preclinical AD using a local weighted regression method (Loess curve). Changes on biomarker levels in CSF are represented as *z*‐scores using Aβ PET in Centiloid scale (CL) as a proxy of disease progression. Abnormal biomarker levels were determined as two standard deviations (SD) above the mean. Incipient Aβ pathology positivity was determined as Aβ PET higher than CL 12 (Mila‐Aloma *et al*, 2020). All samples were run in singlicates.

CSF p‐tau235 had a weak correlation with Aβ PET in the whole ALFA+ cohort (*r*_S_ = 0.32, *P* < 0.0001) (Appendix Fig S5). Further, CSF p‐tau235 had moderate to strong correlation with CSF p‐tau217 (*r*_S_ = 0.67, *P* < 0.0001) and correlated strongly with p‐tau231 (*r*_S_ = 0.80, *P* < 0.0001), highlighting the close relationship between p‐tau235 and p‐tau231 during early AD stages (Appendix Table S4). Finally, we applied a robust local weighted regression method to model the trajectories of each of the CSF tau biomarkers as a function of Aβ PET. CSF p‐tau231 was the first biomarker to surpass the two *z*‐score thresholds above the mean of the A−T− group (used here as definition of abnormality), followed by CSF p‐tau217 and finally p‐tau235 (Fig 5).

### Sequential changes in p‐tau231 and p‐tau235 in preclinical AD: ALFA+ cohort

**Figure 6:**
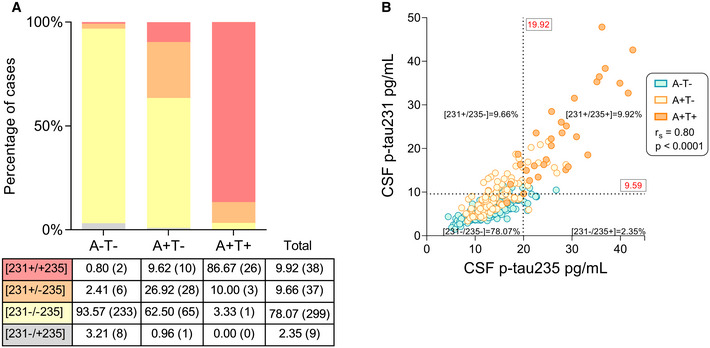
CSF p‐tau231 and p‐tau235 sequential phosphorylation in ALFA+ (preclinical AD) Stacked bar chart depicting the percentages of [231+/235+], [231+/235−], [231−/235−] and [231−/235+] cases in each preclinical group. Table below indicates the exact percentages of participants in each group (exact number of participants in parenthesis). Discordant cases with the sequential phosphorylation hypothesis [231−/+235] are shown in grey.Correlation between CSF p‐tau235 and p‐tau231 in the whole ALFA+ cohort (Spearman’s rank correlation: *r*_S_ = 0.80, *P*˂0.0001). Assay cut‐offs were determined as the mean ± 2 SD of the A−T− group (defining p‐tau231 and p‐tau235 positivity or negativity in each participant). Cut‐off values are indicated in red (19.92 and 9.59 pg/ml for CSF p‐tau235 and p‐tau231, respectively) and displayed with black dashed lines, resulting in four quadrants, each of them representing the four different positive or negatively status for each biomarker. Discordant cases with the sequential phosphorylation hypothesis ([231−/+235]) are shown on the lower right quadrant (2.35% of the total, 9 participants out 383). All samples were run in singlicates.

We investigated the changes in CSF p‐tau231 and p‐tau235 in preclinical AD in the ALFA+ study. Using the mean ± 2 standard deviations (SD) of the A−T− group as a cut‐off, all individuals were classified as [231−/235−], [231+/235−], [231−/235+] and [231+/235+] and plotted in a stacked bar chart in order to illustrate the sequence of phosphorylation in CSF (Fig 6A). In the A−T− group, [231−/235−] cases accounted for 93.57% of the total, whereas the remaining were 2.41% [231+/235−], 0.80% [231+/+235] and 3.21% [231−/+235]. In the A+T− group (early preclinical), 62.50% of the cases were [231−/235−], 26.92% [231+/235−], 9.62% [231+/235+] and only 0.96% [231−/235+]. Finally, in the A+T+ group (late preclinical), the transition to [231+/235+] seemed for the most part completed as 86.67% of the participants were positive for both CSF p‐tau231 and p‐tau235, whereas only 10.00% were [231+/235−]. The remaining 3.33% included exclusively [231−/235−] cases, with no [231−/+235] cases found in A+T+. When analysing the whole ALFA+ cohort, only 2.35% of the participants (9 out of 383) were [231−/+235] (Fig 6A and B). Of note, similar results were obtained when using the 95^th^ percentile cut‐off (Appendix Fig S6A and B).

## Discussion

In this study, we report a novel biomarker discovery, p‐tau235, starting from its quantitative assessment in AD brain tissue, followed by immunoassay development and quantification in CSF. By the combined use of exploratory and targeted IP‐MS approaches, we were able to identify and quantify a double‐phosphorylated tau species containing p‐tau235 in human brain tissue, which we demonstrated to be highly increased in AD. In order to translate these findings from the brain into CSF, we developed a Simoa assay specifically targeting p‐tau235. Subsequently, we measured CSF p‐tau235 in three independent cohorts and demonstrated that (i) CSF p‐tau235 has a high diagnostic accuracy to identify symptomatic AD, similar to CSF p‐tau217 and p‐tau231; (ii) CSF p‐tau235 is significantly increased early in preclinical AD, when only subtle changes in Aβ pathology are detectable in CSF; (iii) CSF p‐tau235 matched the performance of CSF p‐tau231 and outperformed CSF p‐tau217 when discriminating preclinical groups (A+T− vs A+T+); (iv) during the preclinical stage of AD, there is a sequence of changes in p‐tau biomarkers consisting on an initial increase in CSF p‐tau231, followed by an increase in CSF p‐tau235. These changes in CSF are consistent with the sequential phosphorylation described by us in TBS‐soluble AD brain fractions and by others in insoluble PHF material (Hanger *et al*, 2007), ultimately supporting that CSF p‐tau235 could be used to stage the long preclinical phase of AD.

Tau protein in CSF constitutes a pool of soluble fragments of different lengths, primarily comprising protein fragments from the N‐terminal to the proline‐rich region (Sato *et al*, 2018; Cicognola *et al*, 2019; Horie *et al*, 2020). This soluble tau pool originates in the brain presumably through proteolytic processing and represents the source for CSF tau biomarkers. Therefore, we hypothesised that by studying TBS‐soluble fractions of human brain homogenates, we would gain a valuable insight on novel p‐tau species that could serve as fluid biomarkers. Additionally, the brain material has the advantage that it contains large amounts of tau protein, making it suitable for biomarker discovery. Firstly, we used an exploratory IP‐MS approach on AD and control TBS‐soluble fractions, showing that TBS‐soluble fraction does indeed reflect CSF in terms of tau phosphorylation; mono‐phosphorylated tryptic peptides containing p‐tau181, p‐tau217 and p‐tau231 were among the most prominent species identified. In addition, we were able to identify a double‐phosphorylated tryptic peptide containing both p‐tau231 and p‐tau235, which was particularly predominant in AD and virtually absent in the control TBS‐soluble pooled fractions. Subsequently, we developed a targeted IP‐MS approach to quantify mono‐phosphorylated p‐tau231 and double‐phosphorylated p‐tau(231+235) tryptic peptides, this time on individual TBS‐soluble brain fractions from AD and control individuals. Mono‐phosphorylated p‐tau231 tryptic peptide was slightly increased in AD, although this difference was not statistically sustained. In contrast, the double‐phosphorylated p‐tau(231+235) tryptic species were markedly increased in AD, probably due to the intense phosphorylation affecting this threonine/serine cluster during later stages of the disease. Altogether, we demonstrate that p‐tau235 is a highly specific phosphorylation site for AD pathology.

Our findings regarding the importance of p‐tau235 in the development of AD agree with previous studies, where p‐tau231 and p‐tau235 have been found in PHFs (Hasegawa *et al*, 1992; Hoffmann *et al*, 1997; Singer *et al*, 2006; Hanger *et al*, 2007). Additionally, several studies have reported a close relation between these two phosphorylations sites (Goedert *et al*, 1994; Cho & Johnson, 2004; Li & Paudel, 2006; Schwalbe *et al*, 2015). Notably, both p‐tau231 and p‐tau235 have been shown to hinder tubulin assembly, affecting the overall ability of tau to bind microtubules (Sengupta *et al*, 1998; Cho & Johnson, 2004). It must be highlighted that after detailed examination of all MS/MS spectra (from both exploratory and targeted approach), we could not identify any mono‐phosphorylated p‐tau235 tryptic peptide in brain homogenates. P‐tau235 was only detected accompanied by p‐tau231, whereas the latter could also be identified in the mono‐phosphorylated form. Interestingly, it has been reported that phosphorylation in PHFs occurs in a sequential manner along clusters of amino acids throughout the tau sequence (Hanger *et al*, 2007). One of these described clusters includes p‐tau231 and p‐tau235, and in agreement with our findings in TBS‐soluble fraction, p‐tau231 was identified in a mono‐phosphorylated form, whereas p‐tau235 was exclusively detected in combination with p‐tau231 (Hanger *et al*, 2007). Thus, this preceding study, although qualitative and performed on insoluble PHF extracts, aligns with our quantitative findings in TBS‐soluble fractions. Overall, these results suggest that in the brain, the sequential phosphorylation involving p‐tau231 and p‐tau235 likely parallels AD progression, and consequently, p‐tau235 could represent a novel biomarker capable of staging AD. The question is, does this sequential phosphorylation translate into CSF, and if so, does it have potential from a biomarker perspective?

Previous findings suggest that p‐tau235 is measurable in CSF. Early attempts to measure p‐tau species in CSF used antibodies targeting p‐tau231 and p‐tau235 simultaneously (Ishiguro *et al*, 1999; Arai *et al*, 2000; Blennow *et al*, 2001). Our group previously confirmed the presence of p‐tau235 in CSF when validating our in‐house plasma p‐tau231 assay through IP‐MS, showing that the tau peptides captured by the p‐tau231‐specific antibody were either mono‐phosphorylated p‐tau231 or double‐phosphorylated p‐tau(231+235) (preprint: Ashton *et al*, 2021a; Ashton *et al*, 2021b), matching our findings in brain homogenates in the present study. These data, together with our MS results, suggest that p‐tau235 has potential as a fluid biomarker, which led to the development of our in‐house Simoa p‐tau235 assay. Interestingly, when validating our CSF p‐tau235 assay using IP‐MS, we could only identify p‐tau235 accompanied by p‐tau231, once again in a similar fashion as in our brain results. This strengthened the idea of a potential translatability of the sequential phosphorylation into fluid.

Here, we developed a novel immunoassay that measures specifically p‐tau235 in CSF and described its performance in three independent cohorts. In the discovery cohort, we demonstrated that the p‐tau235 assay can readily measure this phosphorylation site in CSF and most notably that CSF p‐tau235 is increased in AD when compared with neurological controls. Next, we proved in the TRIAD cohort that CSF p‐tau235 accurately detects Aβ pathology at different stages of the AD *continuum*. CSF p‐tau235 levels appear to gradually increase from the earlier stages (from CU− to CU+ and MCI+) and then plateau at later stages (only a subtle and not significant increase occurred between MCI+ and AD dementia). This plateau is similar to what has been found for CSF p‐tau217 and p‐tau231 (preprint: Ashton *et al*, 2021a). Interestingly, the increase in CSF p‐tau235 levels began in the asymptomatic AD group (CU+), indicating that CSF p‐tau235 is an early biomarker of AD pathology. It is important to highlight that Aβ positivity in both TRIAD and ALFA+ was determined using the CSF Aβ_1–42/40_ ratio, which is seen as an earlier indicator of amyloid burden than amyloid PET (Palmqvist *et al*, 2017). In order to confirm this finding and further investigate the role of CSF p‐tau235 in preclinical AD, we measured this novel biomarker in the ALFA+ cohort, which is composed exclusively of CU participants. In this cohort, CSF p‐tau235 was mildly but significantly increased already in A+T− participants. Not surprisingly, the increase in CSF p‐tau235 between A+T− and A+T+ was particularly pronounced, as the participants in the latter group are already tau pathology‐positive as measured by CSF p‐tau181.

One of the major breakthroughs in the field of AD biomarkers has been the development of immunoassays capable of measuring p‐tau217 and p‐tau231 in CSF and blood. P‐tau217 has been shown to offer a better performance detecting AD than the widely used p‐tau181 (Janelidze *et al*, 2020; Palmqvist *et al*, 2020). On the other hand, CSF p‐tau231 has previously been reported to be an AD‐specific biomarker (Ishiguro *et al*, 1999; Arai *et al*, 2000; Kohnken *et al*, 2000; Blennow *et al*, 2001), but its performance has often been found to be similar to that of p‐tau181 (Mitchell, 2009). Recently, this view was challenged by a study demonstrating that CSF p‐tau231 is the first emerging p‐tau species in preclinical AD, reaching abnormal levels even before p‐tau217 and p‐tau181 (Suarez‐Calvet *et al*, 2020). The high performance of p‐tau231 identifying early AD pathology was consolidated independently in the TRIAD cohort (preprint: Ashton *et al*, 2021a; Ashton *et al*, 2021b). Therefore, we contextualised the performance of p‐tau235 by comparing it with CSF p‐tau217 and p‐tau231. Our results show that although CSF p‐tau235 increases in preclinical AD, that increase appears to occur later in the AD *continuum* compared with CSF p‐tau231 and p‐tau217. Firstly, CSF p‐tau231 proved superior to CSF p‐tau235 identifying early pathology changes in CSF (CU− *vs* CU+). CSF p‐tau217, on the other hand, was not significantly different from CSF p‐tau235 in this scenario, but its AUC value was higher. This suggests that CSF p‐tau217, similarly to CSF p‐tau231, is superior to CSF p‐tau235 in capturing the earliest AD changes. In contrast, CSF p‐tau235 had a similar accuracy to CSF p‐tau217 and p‐tau231 distinguishing CI− and CI+, which indicates that once cognitive impairment is clinically established, all p‐tau assays perform similarly identifying AD pathology. Secondly, when examining preclinical AD stages in more depth in the ALFA+ cohort, we demonstrated that CSF p‐tau217 and p‐tau231 outperformed CSF p‐tau235 to discriminate A+T− from A−T− presumably because both p‐tau217 and p‐tau231 increase slightly earlier in preclinical AD. Conversely, CSF p‐tau235 had similar accuracies distinguishing A+T+ from A−T− when compared with CSF p‐tau217 and p‐tau231. This again demonstrates that all p‐tau assays perform similarly in identifying AD pathology, if both amyloid and tau pathologies are present. Remarkably, when discriminating between preclinical groups (A+T− vs A+T+), CSF p‐tau235 matched the performance of CSF p‐tau231 and outperformed CSF p‐tau217. Further examination of these data revealed the effect size (as determined by Cohen’s *d*) of the increase in CSF p‐tau235 between A+T− and A+T+ was indeed larger than that of CSF p‐tau231 and p‐tau217. Finally, using Aβ PET as a proxy of disease progression, we modelled the trajectory of CSF p‐tau235 in preclinical AD, situating the emergence of abnormal CSF p‐tau235 values after CSF p‐tau231 and p‐tau217 and before CSF p‐tau181 and total tau. Thirdly, we demonstrated that CSF p‐tau231 and p‐tau235 positivity occurs in a sequential manner during preclinical AD, that is, there is a gradual increase in these phosphorylations across the A−T−, A+T− and A+T+ groups. Importantly, most of the CSF p‐tau235‐positive cases were already CSF p‐tau231 positive, but the opposite was not observed. While 374 out of 383 (97.65%) of the cases concord with the sequential phosphorylation hypothesis in CSF, there were only 9 out of 383 (2.35%, [231−/235+]) discordant cases. These results suggest that the neurpathological findings of a sequential phosphorylation affecting threonine 231 and serine 235 residues in insoluble PHFs (Hanger *et al*, 2007), and as shown in the present study in TBS‐soluble brain fractions, can also be observed in the CSF and may be used as AD biomarkers.

The sequential phosphorylation observed in CSF is particularly relevant as preclinical AD is known to begin approximately 20 years before symptom onset (Sperling *et al*, 2011, 2014). Although this stage in autosomal dominant AD can be studied with the concept of expected years at symptom onset (Bateman *et al*, 2012), preclinical sporadic AD is usually dichotomised between A+T− and A+T+ groups (Suarez‐Calvet *et al*, 2020). Amid this limitation, the combination of CSF p‐tau231 and p‐tau235 may help better delineate this long preclinical stage. CSF p‐tau231 is the earliest p‐tau species abnormally emerging in CSF, and p‐tau235 can only exist preceded by p‐tau231. In other words, p‐tau235 appears to be a very adequate staging biomarker for assessing the later phase of preclinical AD and imminent progression to cognitive decline. For instance, [231+/235−] would indicate an incipient AD pathology (the earliest cases within preclinical AD ‘pre‐amyloid’), whereas [231+/235+] would suggest that individuals have a further degree of AD progression. This result has obvious implications for clinical trials. An intervention aimed at the earliest stages of the disease would include [231+/235−] participants, whereas an intervention in asymptomatic individuals but closer to symptom onset would include [231+/235+] participants.

Our study has some major strengths worth mentioning. Firstly, MS was used to unbiasedly demonstrate that p‐tau235 is a very prevalent posttranslational modification in AD and to confirm the presence of p‐tau235 in CSF. Furthermore, we also used MS to consolidate previous reports suggesting a sequential phosphorylation involving threonine 231 and serine 235 in insoluble PHFs, but this time in TBS‐soluble brain material. Secondly, we developed an in‐house Simoa assay for the quantification of p‐tau235 in CSF, and we investigated this novel biomarker in three independent and well‐characterised cohorts, describing its levels across the AD *continuum*, with a special emphasis on preclinical AD. Thirdly, we compared p‐tau235 with more established p‐tau biomarkers p‐tau217 and p‐tau231 to delineate its strengths and limitations as a biomarker. Finally, we confirmed and extended the results of the discovery cohort in the very comprehensively characterised cohorts TRIAD and ALFA+. On the other hand, this study has some limitations. All CSF cohorts included here are cross‐sectional, and therefore, the results have to be confirmed in longitudinal studies to definitely conclude that p‐tau235 follows the ordinal sequence of phosphorylation. Particularly, a longitudinal cohort would be needed to confirm the progression of [231+/235−] individual into [231+/235+]. Longitudinal cognitive testing would be needed to determine if [231+/235+] at the preclinical stage is a more accurate predictor of future cognitive decline than p‐tau231 alone. Additionally, a clinical cohort comparing the different p‐tau biomarkers would have helped consolidate the performance of p‐tau235 in clinical settings.

In conclusion, this is the first study to demonstrate the role of p‐tau235 with AD in brain tissue and translate this finding into clinical utility, by the detection of p‐tau235 in CSF from individuals at different stages of the AD *continuum* (comprehensively characterised by gold standard CSF and PET biomarkers). We show here that p‐tau235 is a novel and highly specific CSF biomarker for different stages of AD, including preclinical AD. Moreover, combined pathological and CSF data, independently assessed by MS and immunoassays, suggest that the combination of CSF p‐tau235 and p‐tau231 biomarkers may be useful not only to detect preclinical AD but also to stage this long asymptomatic period. Such characterisation could greatly benefit future drug development in clinical trial recruitment, finding the best window to assess the efficacy of candidate compounds or simply evaluating if disease progression has been effectively tackled.

## Materials and Methods

### Post‐mortem human brain tissue

Human post‐mortem brain tissue from frontal grey matter of neuropathology confirmed sporadic AD and control cases was obtained through the brain donation programme at Queen Square Brain Bank for Neurological Disorders (QSBB), Department of Clinical and Movement Neurosciences, Institute of Neurology, University College London (UCL). Sample description and case demographics are presented in Appendix Table S1. All clinical records from the sporadic AD cases used in this study have been screened and no family history was documented. All AD cases have been recruited for brain donation through the Dementia Research Centre, UCL Queen Square Institute of Neurology. AD cases have been screened, and no known mutations causing familial AD have been identified. AD patients fulfilled the 2012 National Institute on Aging and the Alzheimer’s Association (NIA‐AA) guidelines for neuropathological assessment (Hyman *et al*, 2012; Montine *et al*, 2012) and clinical NINCDS criteria for probable AD (McKhann *et al*, 1984). Braak and Braak staging (Braak *et al*, 2006), Thal phases (Thal *et al*, 2002) and CERAD scoring system (Morris *et al*, 1989; Mirra *et al*, 1991), were determined using the appropriate criteria. Samples were stored at −80°C pending homogenisation and biochemical analysis. Human brain tissue was used in accordance with the Helsinki Declaration and the Human Services Belmont Report, and experiments were approved by the regional Ethics Committees at UCL and at the University of Gothenburg.

### Clinical cohorts

Diagnostic performance of the developed Simoa p‐tau235 was evaluated in three different research cohorts:

#### Discovery cohort

The discovery cohort consisted of CSF samples from AD patients (AD, *n* = 19) and neurological controls (control, *n* = 21) (Appendix Table S2). AD patients were accepted for clinical assessment for suspected AD, and core AD CSF biomarkers were measured to ensure a typical AD profile (CSF Aβ_1–42_ < 530 ng/l, p‐tau181 > 60 ng/l and t‐tau > 350 ng/l, all measured with Innotest ELISA). Individuals with other neurological conditions such as cerebrovascular or co‐existing inflammatory disease were excluded. Control individuals consisted of patients with normal core AD CSF biomarkers levels and minor psychiatric and neurological symptoms. Ethical approval for research use of these samples has been granted by the Ethics Committee at the University of Gothenburg (EPN 140811).

#### TRIAD cohort

The cross‐sectional samples reported here belong to the Translational Biomarkers of Aging and Dementia (TRIAD) cohort (McGill University, Canada) and were stratified based on amyloid pathology using CSF Aβ_1–42/40_ (LUMIPULSE G1200, Fujirebio) (Table 1). Participants comprised cognitively unimpaired Aβ pathology negative (CU−, *n* = 50), cognitively unimpaired Aβ pathology positive (CU+, *n* = 32), mild cognitive impairment due to AD (Aβ pathology positive) (MCI+, *n* = 20), AD (*n* = 20) and non‐AD (*n* = 19). This last group included MCI patients negative for Aβ pathology and those clinically diagnosed with frontotemporal dementia. CU participants scored 0 on the Clinical Dementia Rating (CDR) scale, whereas MCI had a CDR score 0.5 and AD had a CDR score greater than 1. MCI patients also presented objective and subjective memory impairments but essentially normal daily living activities. The diagnosis of AD was given according to the National Institute on Aging and the Alzheimer’s Association criteria for probable AD (McKhann *et al*, 2011). Participants with frontotemporal dementia were clinically diagnosed with the behavioural or semantic variant of the disease, with a CDR> 0.5 and negative Aβ PET scan. The core CSF biomarkers for AD were quantified with the LUMIPULSE G1200 (Fujirebio) at the Clinical Neurochemistry Laboratory, Sahlgrenska University Hospital, Mölndal, Sweden, and a CSF Aβ_1–42/40_ cut‐off of 0.068 was used here to determine Aβ pathology status. Additionally, participants were further stratified as cognitively impaired Aβ pathology positive (CI+, including MCI+ and AD) and cognitively impaired Aβ pathology negative (CI−, including exclusively non‐AD) in order to analyse the performance of CSF p‐tau235 once cognitive impairment is established. The TRIAD study was approved by the Research Ethics Board of the Montreal Neurological Institute and the Faculty of Medicine Research Ethics Office (McGill University, Canada). All study participants provided written informed consent.

**Table 1:** Demographics of the 141 participants included in the TRIAD CSF cohort.

TRIAD cohort (*n* = 141)	CU− (*n* = 50)	CU+ (*n* = 32)	MCI+ (*n* = 20)	AD (*n* = 20)	Non‐AD (*n* = 19)	*P*‐value
Females, *n* (%)	31 (62.0)	21 (65.6)	9 (45.0)	9 (45.0)	9 (47.4)	0.35
Age, mean (SD)	70.3 (8.7)#	73.0 (4.2)#	72.2 (7.2) #	64.1 (7.4)*	66.2 (9.7)	˂0.0001
Education years, mean (SD)	15.3 (3.4)	14.0 (3.0)	15.7 (15.9)	15.4 (3.1)	14.4 (3.8)	0.28
*APOE‐ɛ4* carriers, *n* (%)	13 (26)#	12 (37.5)	13 (65)*	13 (65)*	3 (15.8)#	˂0.001
MMSE score, mean (SD)	29.3 (1.0)#	29.1 (0.9)#	28.0 (1.8)#	19.8 (6.2)*	26.8 (5.2)#	˂0.0001
Aβ PET (SUVR), mean (SD)	1.27 (0.13)#	1.82 (0.48)*#	2.39 (2.42)*	2.39 (0.45)*	1.24 (0.12)#	˂0.0001
Tau PET (SUVR), mean (SD)	0.90 (0.10)#	1.00 (0.14)#	1.55 (1.45)*#	2.14 (0.57)*	0.83 (0.11)#	˂0.0001
CSF Aβ_1–42/40_ (pg/ml), mean (SD)	0.09 (0.01)#	0.05 (0.01)*#	0.04 (0.01)*#	0.04 (0.01)*	0.09 (0.01)#	˂0.0001
CSF p‐tau217 (pg/ml), mean (SD)	5.1 (3.1)#	13.7 (16.4)*#	24.3 (10.2)*#	29.1 (22.0)*	4.7 (2.1)#	˂0.0001
CSF p‐tau231 (pg/ml), mean (SD)	9.1 (2.4)#	20.8 (16.3)*#	31.6 (14.1)*#	37.4 (24.7)*	9.1 (3.9)#	˂0.0001
CSF p‐tau235 (pg/ml), mean (SD)	13.1 (3.6)#	17.4 (7.0)*#	26.5 (7.4)*	28.6 (11.0)*	12.7 (3.1)#	˂0.0001

CU, cognitively unimpaired; MCI, mild cognitive impairment; AD, Alzheimer’s disease; MMSE, Mini Mental State Examination; PET, positron emission tomography; SUVR, standardised uptake value ratio; CSF, cerebrospinal fluid; Aβ, amyloid‐beta; SD, standard deviation.

Data are presented as mean (SD). Differences between groups were tested using one‐way ANOVA adjusted by age and sex, followed by Bonferroni‐corrected post‐hoc comparison (continuous variables) or Fisher exact test (categorical variables). Significant differences compared with CU− (*) and AD (#).

#### ALFA+ cohort

The ALFA (for ALzheimer’s and FAmilies) study includes 2.743 middle‐aged (45–74 years old) cognitively unimpaired individuals enriched for a family history of AD. The nested longitudinal ALFA+ study includes 450 participants selected based on their specific AD risk profile, determined by an algorithm in which participants with an AD parental history and *APOE* status, verbal episodic memory score and CAIDE score were taken into consideration. A detailed phenotyping was performed in ALFA+ participants, including a lumbar puncture for the measurement of CSF biomarkers and imaging (MRI and PET) biomarker acquisition. ALFA+ inclusion criteria were (i) individuals who had previously participated in the 45–65/FPM2012 study (ALFA parent cohort); (ii) age between 45 and 65 years at the time of inclusion in the cohort (45–65/FPM2012 study); and (iii) long‐term commitment to the study: inclusion and follow‐up visits and agreement to undergo all tests and study procedures (MRI, PET and lumbar puncture). The ALFA+ exclusion criteria were (i) cognitive impairment (CDR > 0, Mini Mental State Examination [MMSE] < 27 or semantic fluency < 12); (ii) any significant systemic illness or unstable medical condition which could lead to difficulty complying with the protocol; (iii) any contraindication to any test or procedure; and (iv) a family history of monogenic AD. Participants in ALFA+ were stratified based on the presence of Aβ pathology (A+) CSF Aβ_1–42/40_ < 0.071) and tau pathology (T+, CSF p‐tau181 > 24 pg/ml (Mila‐Aloma *et al*, 2020), (Suarez‐Calvet *et al*, 2020)). Core biomarkers were previously measured using the electrochemiluminescence immunoassays Elecsys (p‐tau181 and t‐tau) and the NeuroToolKit (Aβ_1–42_ and Aβ_1–40_) on an automated e 601 Cobas instrument (Roche Diagnostics International Ltd) (Mila‐Aloma *et al*, 2020). CSF p‐tau217 and p‐tau231 have been previously measured with in‐house Simoa assay and ADx immunoassay, respectively (Suarez‐Calvet *et al*, 2020). In the present study, we included the 396 first consecutive ALFA+ participants with available CSF biomarkers. We excluded 13 participants who were Aβ‐negative but tau‐positive (i.e., suspected non‐AD pathological change, A−T+) and hence not within Alzheimer’s *continuum*. Thus, 383 participants from ALFA+ were finally included in the study (Table 2). The ALFA+ study was approved by the Independent Ethics Committee (Parc de Salut Mar, Barcelona, Spain). All study participants provided written informed consent.

**Table 2:** Demographics of the 383 participants included in the ALFA+ CSF cohort.

ALFA+ cohort (*n* = 383)	A−T− (*n* = 248)	A+T− (*n* = 104)	A+T+ (*n* = 31)	*P*‐value
Females, *n* (%)	153 (61.7)	60 (50.7)	21 (67.7)	0.60
Age, mean (SD)	60.5 (4.5)	61.8 (5.0)	63.8 (4.4)*	˂0.0001
Education years, mean (SD)	13.6 (3.5)	13.8 (3.5)	11.9 (3.7)*	0.031
*APOE‐ɛ4* carriers, *n* (%)	105 (42.3)	85 (81.7)*	18 (58.1)	˂0.0001
MMSE score, mean (SD)	29.2 (0.9)	29.2 (0.9)	28.8 (1.1)	0.13
Aβ PET (CL), mean (SD)	−4.5 (6.6)	12.8 (17.2)*	31.9 (27.1)*	˂0.0001
CSF p‐tau181 (pg/ml), mean (SD)	13.9 (4.2)	15.5 (4.1)*	33.4 (11.7)*	˂0.0001
CSF p‐tau217 (pg/ml), mean (SD)	3.9 (2.1)	7.1 (4.2)*	21.1 (12.0)*	˂0.0001
CSF p‐tau231 (pg/ml), mean (SD)	5.9 (1.9)	9.3 (4.1)*	23.2 (10.3)*	˂0.0001
CSF p‐tau235 (pg/ml), mean (SD)	12.5 (3.7)	14.6 (4.4)*	28.2 (7.6)*	˂0.0001
CSF t‐tau (pg/ml), mean (SD)	175 (48.0)	190 (44.8)*	355 (84.7)*	˂0.0001
CSF Aβ_1–42/40_ (pg/ml), mean (SD)	0.09 (0.01)	0.05 (0.01)*	0.04 (0.01)*	˂0.0001

A, amyloid pathology negative (−) / positive (+); T, tau pathology negative (−) / positive (+); MMSE, Mini Mental State Examination; PET, positron emission tomography; CL, Centiloids; CSF, cerebrospinal fluid; Aβ, amyloid‐beta; SD, standard deviation.

Data are presented as mean (SD). Differences between groups were tested using one‐way ANOVA adjusted by age and sex, followed by Bonferroni‐corrected post‐hoc comparison (continues variables) or Fisher exact test (categorical values). Significant differences compared with A−T− (*).

### Protein extraction from human brain

Protein extraction from human brain tissue has been previously described in detail (Camporesi *et al*, 2021). Individual Tris‐buffered saline (TBS)‐soluble fractions of human brain homogenates were used either individually, for quantification with a targeted IP‐MS method (using stable‐isotope labelled internal standards), or as a pool, for exploratory IP‐MS (without stable isotope‐labelled internal standards). AD and control TBS‐soluble pools were generated combining a small volume of each individual TBS‐soluble fraction, using equal amounts of each fraction/case (i.e., 10 µg of total protein from each individual fraction/case).

### Tau immunoprecipitation from human brain and CSF

To immunoprecipitate tau protein from human brain TBS‐soluble fractions (both individual and pooled samples), 4 μg of the monoclonal antibody anti‐tau HT7 (ThermoFisher Scientific, epitope 159–163 [human tau441 numbering]) was conjugated with 50 μl M‐280 Dynabeads (sheep anti‐mouse IgG, Invitrogen) according to the manufacturer’s protocol. HT7‐coated beads were used to immunoprecipitate brain extracts containing 10 μg of total protein. Every sample was brought to a final volume of 1 ml with phosphate‐buffered saline (0.01 M phosphate buffer, 0.14 M NaCl, pH 7.4; PBS) buffer, adding Triton X‐100 to a final concentration of 0.05%, and subsequently incubated overnight at +4°C. Immunoprecipitation from pooled CSF (combining equal volumes of CSF from AD and control cases) was performed using the same aforementioned parameters but with minor protocol modifications. Monoclonal Tau12 (Biolegend, epitope 6–18 [human tau441 numbering]) or polyclonal anti p‐tau235 antibodies were conjugated with M‐280 Dynabeads (sheep anti‐mouse IgG and sheep anti‐rabbit IgG, respectively; Invitrogen). The conjugated beads were added directly to 10 ml of pooled CSF, and every sample was brought to a final concentration of 0.05% Triton X‐100. The samples were incubated overnight at +4°C. In both cases (brain and CSF samples) after incubation, the beads and samples were transferred to a KingFisher magnetic particle processor (ThermoFisher Scientific) for automatic washing and subsequent elution of the bound protein. Elution was achieved with 100 μl of 0.5% formic acid. Eluates were collected, dried in a vacuum centrifuge and stored at −80°C until further use.

### Brain and CSF MS analysis

For the quantitative analysis of targeted IP‐MS in individual TBS‐soluble fractions, 100 fmol of stable isotope‐labelled phosphorylated peptide standards (ThermoFisher Scientific) were added directly to each individual sample. The peptide standards consisted of ^220^TREPKKVAVVR[T]PPKSPSSA(K)SRLQT^245^ and ^220^TREPKKVAVVR[T]PPK[S]PSSA(K)SRLQT^245^, containing one or two phosphorylations (p‐tau231 and p‐tau235 are indicated [T] and [S], respectively). The K240 amino acid was labelled with ^13^C and ^15^N for both peptides as indicated (K). To take into account the efficiency of trypsin digestion, the standards extend five amino acids both N and C terminally of the monitored tryptic peptide, ^225^KVAVVRTPPKSPSSAK^240^.

Both individual and pooled TBS‐soluble brain fractions were trypsin‐digested prior to MS analysis, using 100 ng of trypsin (Promega) in 50 mM ammonium bicarbonate. The samples were brought to a final volume of 50 μl with ammonium bicarbonate and shaken overnight at 100 RPM at +37°C. Trypsination was stopped by adding formic acid to a final concentration of 1%. The samples were dried in a vacuum centrifuge and stored at −80°C pending analysis. No standards were added to immunoprecipitated pooled CSF, which was analysed either trypsin‐digested as previously mentioned or without trypsin (in order to identify endogenous peptides).

Nanoflow LC‐MS analysis was performed similarly, as described previously (Cicognola *et al*, 2019) with some modifications. Briefly, a Dionex 3000 LC‐system (Thermo Fisher Scientific) was coupled to a hybrid quadrupole‐orbitrap Q Exactive (ThermoFisher Scientific) mass spectrometer employing electrospray ionisation. Immunoprecipitated samples, both trypsin‐digested and undigested, were reconstituted in 7 µl of 8% acetonitrile/8% formic acid. For each sample, 6 µl was loaded onto a reverse‐phase Acclaim PepMap C18 trap column (length 20 mm, internal diameter 75 μm, particle size 3 μm, pore size 100 Å, ThermoFisher Scientific) for desalting and sample clean‐up, using a sample loading buffer of 2% acetonitrile/0.05% trifluoroacetic acid at a flow rate of 5 µl/min. Separation was performed with a reversed‐phase Acclaim PepMap C18 analytical column (length 150 mm, internal diameter 75 μm, particle size 2 μm, pore size 100 Å, ThermoFisher Scientific) at a flow rate of 300 nl/min by applying a 50 min long linear gradient from 3 to 40% B; buffer A was 0.1% formic acid, and buffer B was 84% acetonitrile/0.1% formic acid. The mass spectrometer was set to operate in positive ion mode and in a data‐dependent way so that for each full mass scan, up to five MS/MS scans were performed. Fragmentation was performed using higher energy collisional dissociation. Acquisition settings for both full scans and MS/MS scans were resolution 70,000, 1 microscan, target value 10^6^, injection time 250 ms; for MS/MS the normalised collision energy was set at 25. For the quantitative analysis, an inclusion list was used to ensure identification of the ions of interest. LC‐MS data were searched against a custom‐made tau database using Mascot Deamon v2.6/Mascot Distiller v2.6.3 (Matrix Science) for charge and isotope deconvolution prior to the search using the Mascot search engine; see for (Brinkmalm *et al*, 2012) processing settings. Quantitative analysis was performed using Skyline v20.1.0.31 (MacCoss lab) using the LC traces from the full mass scans (identification from MS/MS data ensured the integrity of the data). Examples of MS/MS acquisitions of obtained from brain and CSF samples can be found in Appendix Fig S1.

### Simoa p‐tau235 immunoassay and CSF measurements

Levels of p‐tau235 in CSF were measured in all three previously described cohorts (discovery, TRIAD and ALFA+) with an in‐house developed immunoassay using a Simoa platform on an HD‐X instrument (Quanterix) at the Clinical Neurochemistry Laboratory, Sahlgrenska University Hospital, Mölndal, Sweden. CSF p‐tau235 assay validation is described in detailed in Appendix Supplementary Methods and Appendix Tables S5‐S7. The immunoassay comprises a rabbit polyclonal antibody selective against p‐tau235 (synthesised human tau peptide around the phosphorylation site of Ser235 was used as immunogen, Thermofisher Scientific) coated onto paramagnetic beads and used as capture antibody. Biotin‐conjugated monoclonal antibody targeting the N‐terminal tau region (Tau12, Biolegend) was used as detection antibody. Commercially available recombinant full‐length tau (tau441) in vitro phosphorylated with GSK‐3β (SignalChem) was used as an assay calibrator. Randomised CSF samples were vortexed (30s, 2000 rpm) and diluted 1:2 to Tau 2.0 assay diluent (Quanterix) before measurements. In order to investigate our novel assay and contextualised its performance, CSF p‐tau235 measurements in both TRIAD and ALFA+ cohorts were compared with previously reported CSF p‐tau217 (Simoa) and p‐tau231(ADx Neuroscience ELISA) measurements (Suarez‐Calvet *et al*, 2020; preprint: Ashton *et al*, 2021a). Rabbit polyclonal p‐tau217 (Invitrogen) and mouse monoclonal p‐tau231 (ADx Neuroscience) antibodies were generated against a synthesised human peptide around the phosphorylation Thr217 and Thr231, respectively. Precision and accuracy of CSF p‐tau235 measurements for the cohorts analysed can be found in Appendix Table S3.

### Imaging analysis

#### TRIAD cohort

Participants from the TRIAD cohort underwent an Aβ ([^18^F]AZD4694) and a tau ([^18^F]MK‐6240) PET scan on a Siemens High Resolution Research Tomograph (Siemens Medical Solutions), followed by a T1‐weighted imaging acquisition on a 3T MRI scanner for co‐registration purposes, as previously described (Cselenyi *et al*, 2012; Pascoal *et al*, 2018; Therriault *et al*, 2020). Global tau PET estimation was calculated as the average standardised uptake value ratio (SUVR) of the transentorhinal (Braak stages I‐II) and limbic (Braak stages III‐IV) cortices, and the inferior cerebellum was used as a reference region. SUVRs of [^18^F]MK‐6240 and [^18^F]AZD4694 uptake were acquired for 40–70 min and 90–110 min after injection, respectively. Tau PET positivity was defined as 2.5 SD higher than the mean global SUVR of young participants (SUVR cut‐off 1.03). Individual Braak stage classification was done, as described elsewhere (Pascoal *et al*, 2020). Global Aβ PET estimation was calculated as the average SUVR of the precuneus, cingulate, inferior parietal, medial prefrontal, lateral temporal and orbitofrontal cortices, and the whole cerebellum was used as the reference region. Aβ PET positivity was determined by visual reading by two neurologists blinded to clinical diagnosis.

#### ALFA+ cohort

ALFA+ participants had an Aβ ([^18^F]flutemetamol) PET scan and a T1‐weighted MRI scan acquired within one year of the CSF determinations (82 ± 181 days). A high‐resolution 3D T1‐weighted MRI sequence was acquired in a 3T Philips Ingenia CX scanner (TE/TR = 4.6/9.9 ms, flip angle = 8°; voxel size = 0.75 × 0.75 × 0.75 mm^3^). PET scans were acquired in a Siemens Biograph mCT scanner (Siemens Healthcare, Erlangen, Germany) for 90 min after the administration of a bolus of 185 MBq of [^18^F]flutemetamol, following a cranial computed tomography scan for attenuation correction. PET data were acquired for 20 min, using 4 frames of 5 min, and images were reconstructed in 4 frames × 5 min using 3D ordered subset expectation maximisation algorithm (8 iterations, 21 subsets) by incorporating time of flight and point spread function modelling. PET images were processed following a validated Centiloid pipeline using SPM12 (Klunk *et al*, 2015; Salvado *et al*, 2019). Centiloid values were calculated from the mean values of the standard Centiloid target region using the previously calibrated transformation (Salvado *et* *al*, 2019).

### Statistics

SPSS (v26, IBM, Armonk, NY), MedCalc (Ostend, Belgium) and R programming language were used for statistical analysis. Visualisations were generated with GraphPad Prism (v7.03, San Diego, California, USA). Box plots display the median and the interquartile range; whiskers show the 25^th^ and 75^th^ percentiles. Parametric and non‐parametric tests were used when pertinent, depending on the normality of the data. Group comparisons were tested with Mann–Whitney (two categories) and one‐way ANOVA adjusted by age and sex, followed by Bonferroni‐corrected post‐hoc pairwise comparisons (multiple categories). The size effect of group differences was determined using Cohen d (*d*) adjusted by age and sex. Associations between biomarkers were assessed using Spearman’s rank correlation (*r*_S_). The specificity and sensitivity of p‐tau235 assay were determined based on the area under the curve (AUC) of receiver operating characteristics (ROC) analysis. The 95% confidence interval of AUC is abbreviated as CI_95%_ (not to be confused with cognitive impairment, CI). Comparisons between AUC values of different biomarkers were performed using DeLong test package (MedCalc). Sequential phosphorylation hypothesis involving p‐tau231 and p‐tau235 in CSF was evaluated using two different cut‐off methods to determine biomarker positivity/negativity: (i) mean ± 2 SD and (ii) 95^th^ percentile, both calculated in A‐T‐ group (ALFA+). The cut‐offs were subsequently used for defining the samples into four categories: [231−/235−], [231+/235−], [231−/235+] and [231+/235+]. [231−/235+] cases were considered discordant with the sequential phosphorylation hypothesis (Hanger *et al*, 2007). The percentage of concordance in the whole cohort was determined as 100% minus the percentage of discordant cases [231−/235+].

## Author contributions

JL‐R, AS, GB, MS‐C, PRN, HZ and KB created the concept and design. Data acquisition was performed by JL‐R, AS, EC, LG‐M and GB. JL‐R, AS, AB, MM‐A, GB and MS‐C performed data analysis. JL‐R, AS, ALB, MM‐A, EC, LM‐G, NJA, AV, TKK, JDG, GS, MS, CET, TLL, HZ, MS‐C, GB, PRN and KB contributed to sample selection and/or interpretation of data. JL‐R and AS drafted the manuscript, and all authors revised it. All authors read and approved the final manuscript.

## Conflict of interest

HZ has served at scientific advisory boards and/or as a consultant for Alector, Eisai, Denali, Roche Diagnostics, Wave, Samumed, Siemens Healthineers, Pinteon Therapeutics, Nervgen, AZTherapies, CogRx and Red Abbey Labs; has given lectures in symposia sponsored by Cellectricon, Fujirebio, Alzecure and Biogen; and is a co‐founder of Brain Biomarker Solutions in Gothenburg AB (BBS), which is a part of the GU Ventures Incubator Program. KB has served as a consultant, at advisory boards, or at data monitoring committees for Abcam, Axon, Biogen, JOMDD/Shimadzu. Julius Clinical, Lilly, MagQu, Novartis, Prothena, Roche Diagnostics, and Siemens Healthineers, and is a co‐founder of Brain Biomarker Solutions in Gothenburg AB (BBS), which is a part of the GU Ventures Incubator Program. MSC has served as a consultant and at advisory boards for Roche Diagnostics International Ltd and has given lectures in symposia sponsored by Roche Diagnostics, S.L.U and Roche Farma, S.A.

## Supporting information



Appendix

## Data Availability

Bulk anonymised data can be shared by request from qualified investigators, providing data transfer is in agreement with EU legislation and decisions by the IRB of each participating centre. Data and/or samples from TRIAD and ALFA+ can be requested on https://datashare.tnl‐mcgill.com/ds/ and https://www.gaaindata.org/ respectively.
