# Supplementary material for: P‐tau235: a novel biomarker for staging preclinical Alzheimer’s disease
Source: EMBO Mol Med. 2021 Nov 2;13(12):e15098. doi: 10.15252/emmm.202115098 (PMC8649868; doi:10.15252/emmm.202115098)
Supplement: Supplementary file 1 — Appendix [file EMMM-13-e15098-s002.pdf]

P-tau235: a novel biomarker for staging preclinical Alzheimer's disease

Juan Lantero-Rodriguez[1*], Anniina Snellman[1,2*], Andrea L. Benedet [1,3], Marta Milà-Alomà [4,5,6,7], Elena Camporesi[1], Laia Montoliu-Gaya[1], Nicholas J. Ashton[1,8,9,10], Agathe Vrillon[11,12], Thomas K. Karikari[1,13], Juan Domingo Gispert[4,5,14], Gemma Salvadó[4,5], Mahnaz Shekari[4,5,7], Christina E. Toomey[15,16], Tammaryn L. Lashley[15,16], Henrik Zetterberg[1,16,17,18,19], Marc Suárez-Calvet [4,5,6,20], Gunnar Brinkmalm[1], Pedro Rosa Neto[21,22], Kaj Blennow[1,17,18]

[1]Department of Psychiatry and Neurochemistry, Institute of Neuroscience & Physiology, the Sahlgrenska Academy at the University of Gothenburg, Mölndal, Sweden; [2]Turku PET Centre, University of Turku, Turku, Finland; [3]Translational Neuroimaging Laboratory, McGill Centre for Studies in Aging, McGill University, Montreal, QC, Canada; [4]Barcelonaβeta Brain Research Center (BBRC), Pasqual Maragall Foundation, Barcelona, Spain; [5]Hospital del Mar Medical Research Institute (IMIM), Barcelona, Spain; [6]Centro de Investigación Biomédica en Red de Fragilidad y Envejecimiento Saludable (CIBERFES), Madrid, Spain; [7]Universitat Pompeu Fabra, Barcelona, Spain; [8]Wallenberg Centre for Molecular and Translational Medicine, University of Gothenburg, Gothenburg, Sweden; [9]Department of Old Age Psychiatry, Maurice Wohl Clinical Neuroscience Institute, King's College London, London, UK; [10]NIHR Biomedical Research Centre for Mental Health & Biomedical Research Unit for Dementia at South London & Maudsley NHS Foundation, London, UK; [11]Université de Paris, Cognitive Neurology Center, GHU Nord APHP Hospital Lariboisière Fernand Widal, Paris, France; [12]Université de Paris, Inserm UMR S11-44 Therapeutic Optimization in Neuropsychopharmacology, Paris, France; [13]Department of Psychiatry, University of Pittsburgh, Pittsburgh, PA, USA.; [14]Centro de Investigación Biomédica en Red Bioingeniería, Biomateriales y Nanomedicina (CIBER-BBN), Madrid, Spain; [15]The Queen Square Brain Bank for Neurological Disorders, Department of Clinical and Movement Neurosciences, UCL Institute of Neurology, London, UK; [16]Department of Neurodegenerative Disease, UCL Institute of Neurology, University College London, Queen Square, London, UK; [17]Clinical Neurochemistry Laboratory, Sahlgrenska University Hospital, Mölndal, Sweden; [18]UK Dementia Research Institute at UCL, London, UK; [19]Hong Kong Center for Neurodegenerative Diseases, Hong Kong, China; [20]Servei de Neurologia, Hospital del Mar, Barcelona, Spain; [21]Montreal Neurological Institute, Montreal, QC, Canada;[22]Department of Neurology and Neurosurgery, McGill University, Montreal, QC, Canada.

*Contributed equally as first authors

Corresponding author: Juan Lantero Rodriguez, MSc, Institute of Neuroscience and Physiology, Department of Psychiatry and Neurochemistry, University of Gothenburg, Mölndal, Sweden

Tel: +46 073-715 67 46

Email: juan.rodriguez.2@gu.se

TABLE OF CONTENTS

1. APPENDIX METHODS

Analytical validation of CSF p-tau235 Simoa assay.

2. APPENDIX TABLES

Appendix Table S1. Full case demographics for brain cohort

Appendix Table S2. Demographics of the discovery cohort

Appendix Table S3. Precision and accuracy (cohorts)

Appendix Table S4. Spearman's rank correlation table of CSF p-tau235 with other biomarkers

Appendix Table S5. Dilution linearity (assay validation)

Appendix Table S6. Spike recovery (assay validation)

Appendix Table S7. Precision and accuracy (assay validation)

3. APPENDIX FIGURES

Appendix Figure S1. Examples of MS/MS acquisitions in CSF and brain

Appendix Figure S2. Mass spectrometric validation of CSF p-tau235 assay (assay validation)

Appendix Figure S3. CSF p-tau235 correlation with Aβ and tau PET in TRIAD cohort

Appendix Figure S4. CSF p-tau235 measurements across all Braak stages in TRIAD cohort

Appendix Figure S5. CSF p-tau235 correlation with Aβ PET in ALFA+ cohort

Appendix Figure S6. Sequential phosphorylation in CSF: p-tau231 and p-tau235

1. APPENDIX METHODS

Analytical validation of CSF p-tau235 assay

CSF p-tau235 assay validation included dilution linearity, spike recovery, quantification limits, precision and accuracy.

Dilution linearity was determined performing a 2-fold dilution series (1:2, 1:4, 1:8, 1:16, 1:32, 1:64) on two human CSF samples and one diluent sample spiked with 70 pg/mL of GSK3-β full-length tau-441 (Appendix Table S5). Percentage of recovery (% Recovery) was calculated using the following formula:

$$\% \, Recovery = \frac{Concentration \ at \ dilution_{1:2} \ x \ Dilution \ factor_{1:2}}{Concentration \ at \ dilution_{1:y} \ x \ Dilution \ factor_{1:y}} \ x \ 100$$

Spike recovery, two different CSF samples were diluted 1:2 and spiked with 50pg/mL of GSK-3-β phosphorylated full length tau and compared with the non-spiked CSF. Expected concentration was determined as the measured concentration of the unspiked samples plus the concentration of the spike added (Appendix Table S6). Percentage of spike recovery (% Spike recovery) was calculated using the following formula:

$$\% \, Spike \ recovery \ = \frac{Concentration_{spiked \ sample} - Concentratio \ _{unspiked \ sample}}{Concentration_{added}} \ x \ 100$$

Quantification limits consisted on limit of detection (LOD) and lower limit of quantification (LLOQ). LOD was calculated by running 16 blanks (assays diluent) and adding 3 standard deviations (SD) to the mean value. LOD = 0.03 pg/mL. LLOQ was calculated by running 16 blanks (assays diluent) and adding 10 standard deviations (SD) to the mean value. LLOQ = 0.51 pg/mL

Precision and accuracy was evaluated using two CSF human samples, which were loaded on the plates in replicates of five and run individually as duplicates during three consecutive days, calculating both inter-assay precision (CV_{RW} (%)) and intra-assay precision (CV_R (%)) (Appendix Table S7).

Mass spectrometric validation of the antibodies used in the assays was performed as described in "Methods" section. In summary, the specificity of capture and detector antibodies to bind their respective targets was confirmed performing an exploratory IP-MS approach in CSF pool (Appendix Figure S1 and S2).

APPENDIX TABLES

Appendix Table S1. Full case demographics for brain cohort (frontal grey matter, FGM).

Diagnosis	Sex	AAO (years)	AAD (years)	PM delay (h)	Brain Weight (g)	ApoE genotype	CAA	Thal Phase	Braak staging	CERAD score	ABC score
Control (FGM)	M	na	101	60.3	1450	ε2/ε3	1	0	I	0	A0B1C0
	M	na	38	80.4	1581	ε3/ε4	0	0	0	0	A0B0C0
	F	na	86	119.1	1230	ε3/ε4	0	4	I	1	A3B1C1
	F	na	87	51.4	1114	ε3/ε3	0	1	I	0	A1B1C1
	F	na	86	40.2	1238	ε3/ε3	0	0	0	0	A0B0C0
	F	na	78	29.3	1225	ε2/ε2	0	1	I	0	A0B1C0
	F	na	68	45.1	1330	ε2/ε3	0	0	0	0	A0B0C0
	M	na	69	171	1435	ε3/ε3	1	3	I	1	A2B1C1
	F	na	79	88.5	1288	ε3/ε3	0	2	I	1	A2B1C1
	M	na	95	89.4	1346	ε2/ε3	0	2	I	1	A2B1C1
AD (FGM)	M	54	65	34.3	1089	ε4/ε4	3	5	VI	3	A3B3C3
	M	48	63	31.4	1042	ε3/ε3	3	5	VI	3	A3B3C3
	M	44	59	90.5	1338	na	3	5	VI	3	A3B3C3
	F	69	76	42	1236	na	0	5	VI	2	A3B3C2
	M	71	86	95.10	1203	ε3/ε3	3	5	V	3	A3B3C3
	M	54	67	32.15	1458	ε4/ε4	3	5	VI	3	A3B3C3
	F	49	69	40.1	986	ε4/ε4	3	5	VI	2	A3B3C2
	M	52	71	45.4	1097	ε3/ε3	3	5	VI	3	A3B3C3
	F	58	62	92.2	1234	ε3/ε4	1	5	VI	2	A3B3C2
	F	57	76	57.5	1303	ε3/ε4	2	5	VI	2	A3B3C2

Abbreviations: sex (M = male, F = female), AAO = age at onset, AAD = age at death, PM delay = post-mortem delay, CERAD = consortium to establish a registry for Alzheimer´s disease, CAA = cerebral amyloid angiopathy, The ABC score is a composite of three different assessments, and it incorporates (A) Thal phases of amyloid deposition, (B) Braak stage of neurofibrillary tangles and (C) score of amyloid neuritic plaques (CERAD). na = non-available.

Appendix Table S2. Demographics of the discovery cohort.

Discovery cohort (n = 40)	AD (n = 19)	Control (n = 21)	*P*-value
Females, n (%)	14 (73.68%)	7 (33.33%)	0.012*
Age, mean (SD)	78 (6.03)	70.19 (8.31)	0.003*
CSF A$\beta_{1\text{-}42}$ (pg/ml), mean (SD)	527 (104)	933 (157)	<0.0001*
CSF t-tau (pg/ml), mean (SD)	801 (449)	297 (87)	<0.0001*
CSF p-tau181 (pg/ml), mean (SD)	100 (41)	51 (12)	<0.0001*
CSF p-tau235 (pg/mL), mean (SD)	18.2 (9.8)	7.2 (2.4)	<0.0001*

Abbreviations: AD, Alzheimer's disease; CU, cognitively unimpaired; CSF, cerebrospinal fluid; Aβ, amyloid-beta. Data is presented as mean (standard deviation, SD). Differences between groups were tested using with Mann-Whitney U-test (continues variables) or Fisher exact test (categorical values). Significant differences: P <0.05 (*)

Appendix Table S3. Precision and accuracy (cohorts).

Cohort	Sample	Concentration (pg/ml)	Repeatability (CVr) %	Intermediate precision (CVRw) %
TRIAD cohort	Low QC	13	5.8	5.8
	High QC	29	8.7	8.7
	Mean	-	7.3	7.3
ALFA+ cohort	Low QC	17	4.1	4.1
	High QC	37	12.6	12.8
	Mean	-	8.4	8.5

Abbreviations: QC, quality controls.

Appendix Table S4. Spearman's correlations of p-tau235 with other biomarkers.

Cohort	CSF A$\beta_{1\text{-}42}$	CSF A$\beta_{1\text{-}42/40}$	CSF t-tau	CSF p-tau181	CSF p-tau217	CSF p-tau231
Discovery						
Whole cohort (n = 40)	-0.77 (*P* <0.0001)*	-	0.86 (*P* <0.0001)*	0.78 (*P* <0.0001)*	-	-
Control (n = 21)	-0.17 (*P* = 0.46)	-	0.57 (*P* = 0.007)*	0.37 (*P* = 0.099)	-	-
AD (n = 19)	-0.21 (*P* = 0.39)	-	0.66 (*P* = 0.002)*	0.66 (*P* = 0.002)*	-	-
TRIAD						
Whole cohort (n = 141)	-	-0.67 (*P* <0.0001)*	-	-	0.87 (*P* <0.0001)*	0.89 (*P* <0.0001)*
CU- (n = 50)	-	-0.06 (*P* = 0.67)	-	-	0.62 (*P* = 0.001)*	0.81 (*P* <0.0001)*
CU+ (n = 32)	-	-0.48 (*P* = 0.006)*	-	-	0.76 (*P* <0.0001)*	0.75 (*P* <0.0001)*
MCI+ (n = 20)	-	-0.71 (*P* = 0.001)*	-	-	0.93 (*P* <0.0001)*	0.91 (*P* <0.0001)*
AD (n = 20)	-	-0.31 (*P* = 0.19)	-	-	0.72 (*P* <0.0001)*	0.84 (*P* <0.0001)*
Non-AD (n = 19)	-	-0.24 (*P* = 0.32)	-	-	0.80 (*P* <0.0001)*	0.76 (*P* <0.0001)*
ALFA+						
Whole cohort (n = 383)	-	-0.23 (*P* <0.0001)*	0.81 (*P* <0.0001)*	0.81 (*P* <0.0001)*	0.67 (*P* <0.0001)*	0.80 (*P* <0.0001)*
A-T- (n = 248)	-	0.31 (*P* <0.0001)*	0.77 (*P* <0.0001)*	0.76 (*P* <0.0001)*	0.51 (*P* <0.0001)*	0.76 (*P* <0.0001)*
A+T- (n = 104)	-	-0.36 (*P* <0.0001)*	0.71 (*P* <0.0001)*	0.76 (*P* <0.0001)*	0.70 (*P* <0.0001)*	0.76 (*P* <0.0001)*
A+T+ (n = 31)	-	-0.38 (*P* = 0.035)*	0.62 (*P* <0.0001)*	0.76 (*P* <0.0001)*	0.83 (*P* <0.0001)*	0.83 (*P* <0.0001)*

Abbreviations: CSF, cerebrospinal fluid; Aβ, amyloid-beta; CU, cognitively unimpaired; AD, Alzheimer's disease, MCI, mild cognitive impairment; A, amyloid pathology negative (-) / positive (+); T, tau pathology negative (-) / positive (+). A$\beta_{1\text{-}42}$, t-tau and p-tau181 in discovery cohort were measured using Innostest ELISA. A$\beta_{1\text{-}42/40}$ in TRIAD cohort was measured using Lumipulse (Fujirebio). A$\beta_{1\text{-}42/40}$, t-tau and p-tau181 in ALFA+ were measured using Elecsys (RocheDiagnostics). Significant correlations (P <0.05) are indicated with an asterisk (*).

Appendix Table S5. Dilution linearity (assay validation).

Dilution	CSF 1 (pg/ml)	% Recovery in CSF 1	CSF 2 (pg/ml)	% Recovery in CSF 2	Spiked Diluent (pg/ml)	% Recovery in spiked diluent
1:2	65.6	-	73.9	-	73.6	
1:4	105.8	62.0	106.26	69.5	73.0	100.8
1:8	119.5	54.9	125.4	58.9	66.8	110.3
1:16	132.2	49.6	137.6	53.7	78.0	94.3
1:32	137.7	47.8	132.2	55.9	69.9	105.3
1:64	125.4	52.3	137.8	53.6	84.3	87.4

Appendix Table S6. Spike recovery (assay validation).

<table>
<tr><th colspan="2">Sample</th><th>Measured x Df (pg/mL)</th><th>Mean (pg/mL)</th><th>% CV conc.</th><th>Expected conc. (pg/mL)</th><th>Spike recovery (%)</th></tr>
<tr><td rowspan="4">CSF1</td><td rowspan="2">Neat</td><td>23.3</td><td rowspan="2">23.5</td><td rowspan="2">1.9</td><td rowspan="2">-</td><td rowspan="2">-</td></tr>
<tr><td>23.7</td></tr>
<tr><td rowspan="2">Spike</td><td>70.9</td><td rowspan="2">70.0</td><td rowspan="2">1.2</td><td rowspan="2">73.5</td><td rowspan="2">93.0</td></tr>
<tr><td>69.0</td></tr>
<tr><td rowspan="4">CSF2</td><td rowspan="2">Neat</td><td>22.8</td><td rowspan="2">23.3</td><td rowspan="2">3.1</td><td rowspan="2">-</td><td rowspan="2">-</td></tr>
<tr><td>23.8</td></tr>
<tr><td rowspan="2">Spike</td><td>78.0</td><td rowspan="2">78.8</td><td rowspan="2">1.4</td><td rowspan="2">73.3</td><td rowspan="2">111.1</td></tr>
<tr><td>79.6</td></tr>
</table>

Abbreviations: Df, dilution factor.

Appendix Table S7. Precision and accuracy (assay validation).

Sample	Conc (pg/ml)	Repeatability (CVr) %	Intermediate precision (CVRw) %
CSF1	17	2.6	16.3
CSF2	43	2.7	14.9
Mean	-	2.7	15.6

APPENDIX FIGURES

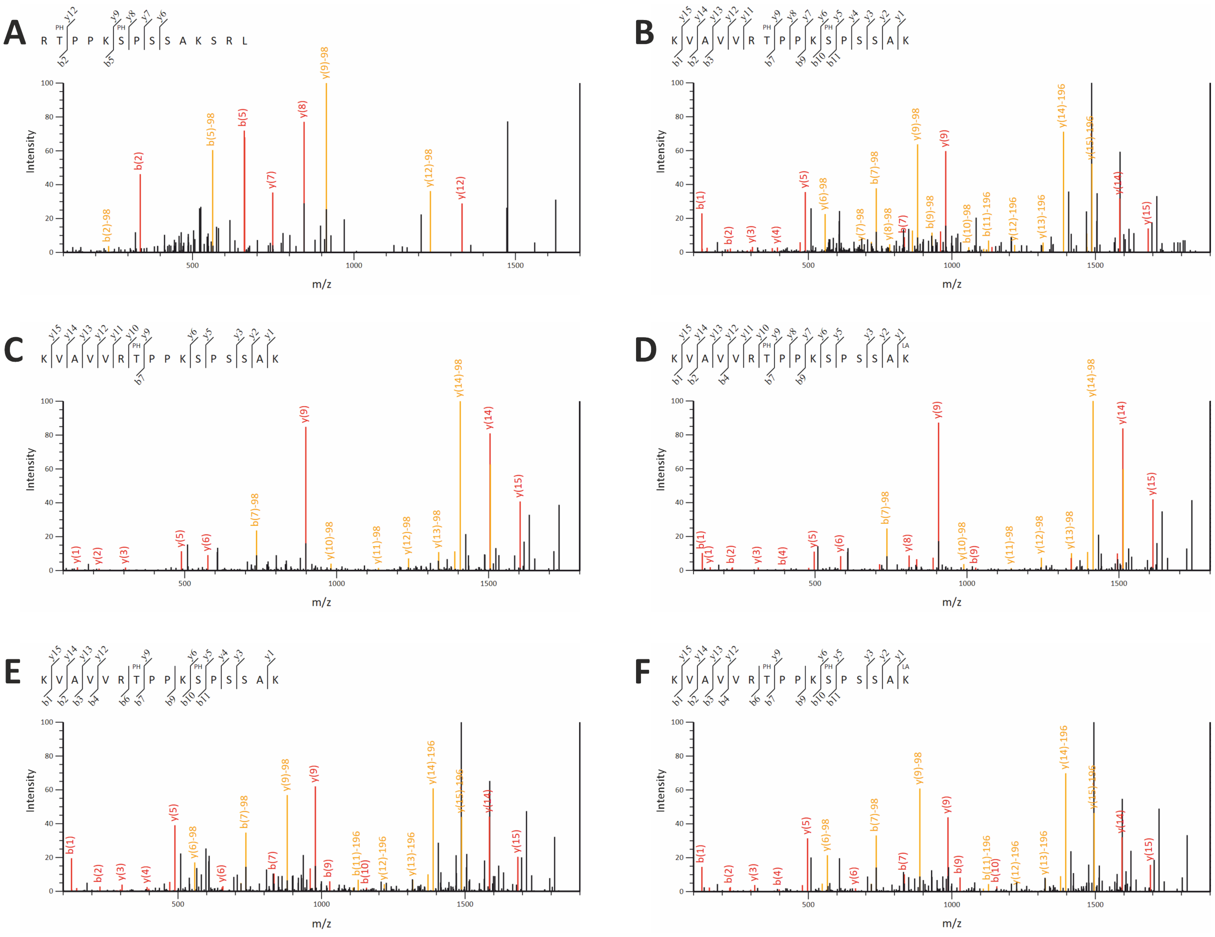

Appendix Figure S1. Examples of MS/MS acquisitions of phosphorylated endogenous and tryptic tau peptides obtained for assay validation in CSF and brain. Peaks matched against theoretical fragments are in red or orange, where -98 indicates additional loss of PO_4. The endogenous peptide detected is tau 230-243, while the tryptic peptides detected are tau 225-240. The stable-isotope labelled internal standard (IS) peptide used for quantification is labelled with $^{13}C^{15}N$ at K240. (A) Endogenous and (B) tryptic peptides containing phosphorylations at both p-tau231 and p-tau235 detected in CSF after performing IP with an anti-tau p-tau235 antibody. (C, D) MS/MS acquisitions obtained with IP-MS showing mono-phosphorylated p-tau231 tryptic peptide detected in the TBS-soluble fraction of brain (C) and of the IS used in the quantification this peptide (D). (E, F) MS/MS acquisitions obtained with IP-MS showing the doubly phosphorylated p-tau(231+235) tryptic peptide quantified in TBS-soluble brain fractions (E) and of the IS used in the quantification this peptide (F).

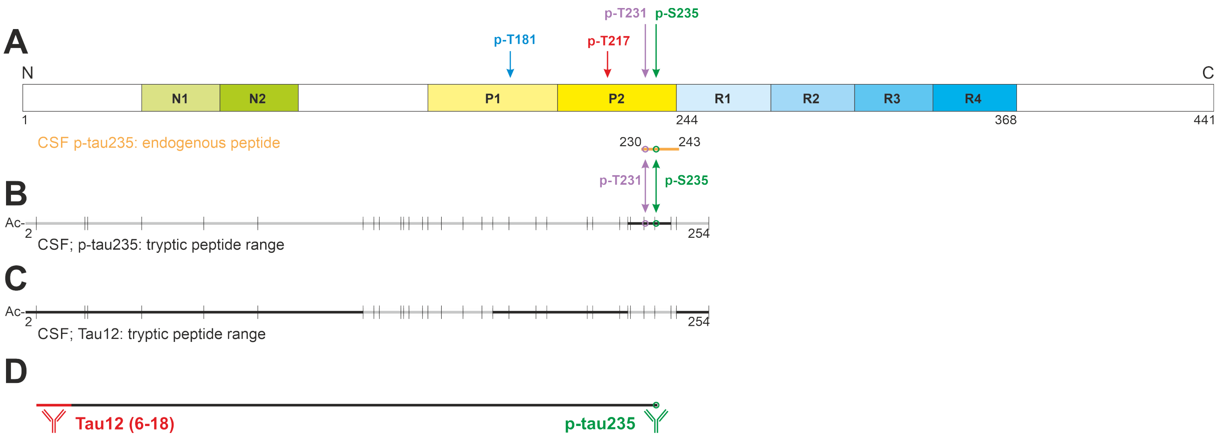

Appendix Figure S2. **Mass spectrometric validation of CSF p-tau235 assay (assay validation).** (A) Schematic illustration depicting full-length tau-441. N-terminal inserts are represented in green (N1 and N2), proline-rich region in yellow (P1 and P2) and MTBR inserts in blue (R1, R2. R3 and R4). Location of prominent phosphorylations is indicated with arrows (p-tau181 in blue, p-tau217 in red, p-tau231 in purple and p-tau235 in green). In orange, endogenous tau peptides identified in human CSF after IP-MS using anti-p-tau235 antibody (used as capture in the assay). Endogenous peptide discovered: doubly phosphorylated 230-243 p-tau(231+p-tau235). (B) Tryptic tau peptides identified in human CSF after IP-MS using anti-p-tau235 antibody, superimposed on *in silico* tau tryptic peptide range. Tryptic peptide discovered: doubly phosphorylated 225-240 p-tau(231+p-tau235). (C) Tryptic tau peptides identified in human CSF after IP-MS using Tau12 antibody (used as detector in the assay), superimposed on *in silico* tau tryptic peptide range. Note that the tryptic range expanded from amino acid 1 to 254. (D) Schematic description of the Simoa p-tau235 assay, showing the minimum peptide length necessary for detection and quantification.

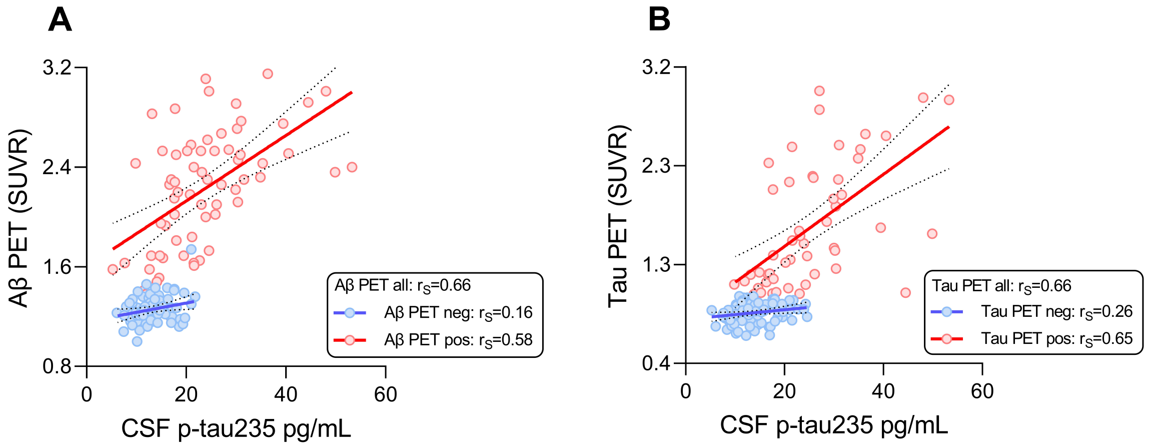

Appendix Figure S3. **CSF p-tau235 Spearman's rank correlation with Aβ and tau PET in TRIAD cohort.** Participants positivity and negativity for Aβ and tau PET is depicted in red and blue respectively. (A) CSF p-tau235 correlation with Aβ PET. Aβ PET all: $r_S = 0.66$ ($P < 0.0001$), Aβ PET negative: $r_S = 0.16$ ($P = 0.17$), Aβ PET positive: $r_S = 0.58$ ($P < 0.0001$). (B) CSF p-tau235 correlation with tau. PET Tau PET all: $r_S = 0.66$ ($P <$ 0.0001), tau PET negative: $r_S = 0.26$ ($P = 0.015$) tau PET positive: $r_S = 0.65$ ($P < 0.0001$).

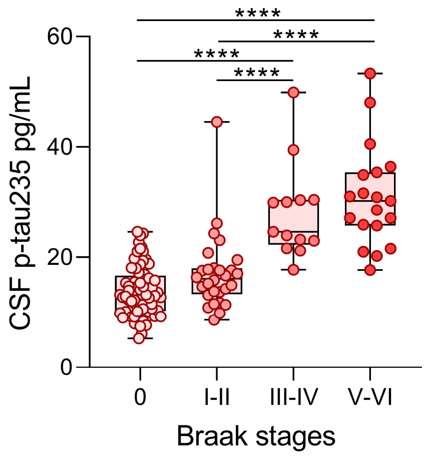

Appendix Figure S4. CSF p-tau235 concentrations across the different Braak stages (TRIAD cohort). *P*-values determined using one-way ANOVA adjusted by age and sex followed by Bonferroni-corrected post hoc pairwise comparisons (*, *P*<0.05; **, *P*<0.01; *** *P*<0.001, **** *P*<0.0001).

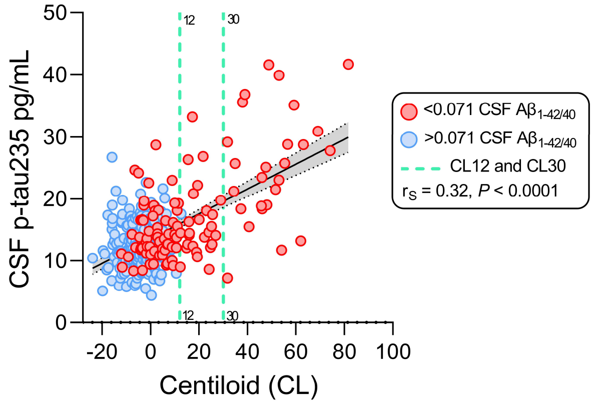

Appendix Figure S5. **CSF p-tau235 Spearman's rank correlation with Aβ PET (in centiloid scale, CL) in ALFA+ cohort.** Participant positivity or negative for CSF $A\beta_{1\text{-}42/40}$ is depicted in red and blue respectively. Green dashed lines indicate CL12 and CL30. Correlation with Aβ PET in the whole cohort: $r_s = 0.32$ (*P* <0.0001). Using CL30 as a cut-off for Aβ PET positivity: r_s (negative) = 0.18 (*P* = 0.0015), r_s (positive) = 0.30 (*P* = 0.1330). Using CL12 as a cut-off for Aβ PET positivity: r_s (negative) = 0.12 (*P* = 0.0765), r_s (positive) = 0.53 (*P* <0.0001).

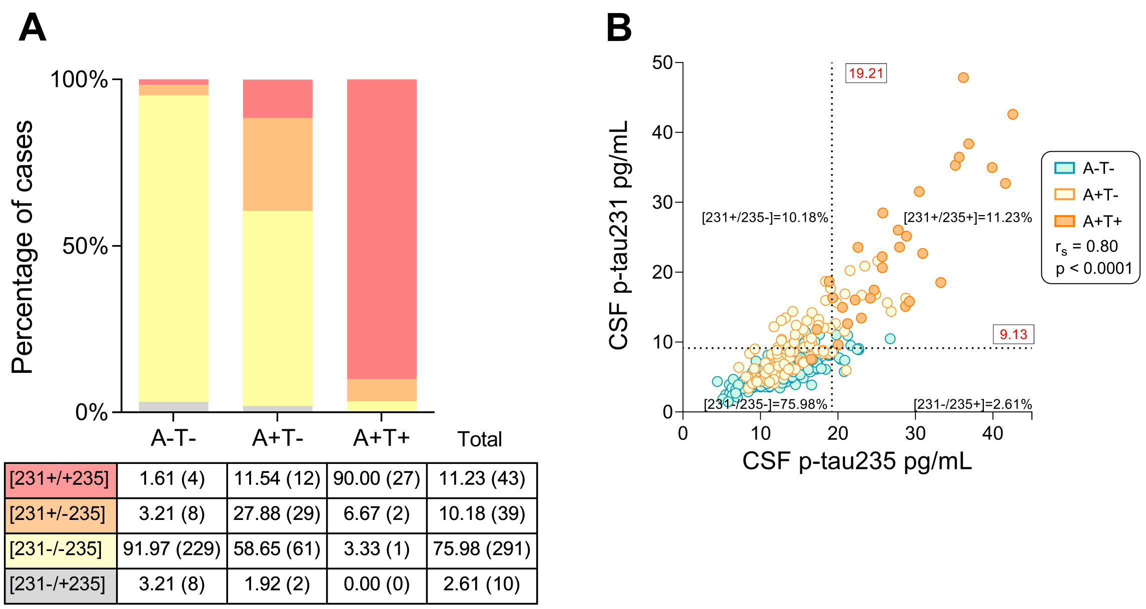

	A-T-	A+T-	A+T+	Total
[231+/+235]	1.61 (4)	11.54 (12)	90.00 (27)	11.23 (43)
[231+/-235]	3.21 (8)	27.88 (29)	6.67 (2)	10.18 (39)
[231-/-235]	91.97 (229)	58.65 (61)	3.33 (1)	75.98 (291)
[231-/+235]	3.21 (8)	1.92 (2)	0.00 (0)	2.61 (10)

Appendix Figure S6. CSF p-tau231 and p-tau235 sequential phosphorylation in ALFA+ (preclinical AD). (A) Stacked bar chart depicting the percentages of [231+/235+], [231+/235-], [231-/235-] and [231-/235+] cases in each preclinical group. Table below indicates the exact percentages of participants in each group (exact number of participants in parenthesis). Discordant cases with the sequential phosphorylation hypothesis [231-/+235] shown in grey. (B) Correlation between CSF p-tau235 and p-tau231 in the whole ALFA+ cohort (Spearman's rank correlation: r_S = 0.80, *P*<0.0001). Assay cut-offs were determined as the *95th percentile* of the A-T- group (defining p-tau231 and p-tau235 positivity or negativity in each participant). Cut-off values are indicated in red (19.21 and 9.13 pg/mL for CSF p-tau235 and p-tau231 respectively) and displayed with black dashed lines, resulting in four quadrants, each of them representing the four different positive or negatively status for each biomarker. Discordant cases with the sequential phosphorylation hypothesis ([231-/+235]) showed on the lower right quadrant (2.61% of the total, 10 participants out 383). Abbreviations: A, amyloid pathology negative (-) / positive (+); T, tau pathology negative (-) / positive (+).
