# Supplementary material for: P‐tau235: a novel biomarker for staging preclinical Alzheimer’s disease
Source: EMBO Mol Med. 2021 Nov 2;13(12):e15098. doi: 10.15252/emmm.202115098 (PMC8649868; doi:10.15252/emmm.202115098)
Supplement: Supplementary file 2 [file EMMM-13-e15098-s001.pdf]

EMBO Molecular Medicine

P-tau235: a novel biomarker for staging preclinical Alzheimer's disease

Juan Lantero-Rodríguez, Anniina Snellman, Andréa L. Benedet, Marta Milà-Alomà, Elena Camporesi, Laia Montoliu-Gaya, Nicholas Ashton, Agathe Vrillon, Thomas Karikari, Juan Domingo Gispert, Gemma Salvadó, Mahnaz Shekari, Christina Toomey, Tammaryn Lashley, Henrik Zetterberg, Marc Suárez-Calvet, Gunnar Brinkmalm, Pedro Rosa-Neto, and Kaj Blennow
DOI: 10.15252/emmm.202115098

Corresponding author(s): Juan Lantero Rodríguez (juan.rodriguez.2@gu.se)

Review Timeline:

Submission Date:	6th Sep 21
Editorial Decision:	23rd Sep 21
Revision Received:	6th Oct 21
Editorial Decision:	13th Oct 21
Revision Received:	14th Oct 21
Accepted:	18th Oct 21

Editor: Jingyi Hou

Transaction Report:

(Note: With the exception of the correction of typographical or spelling errors that could be a source of ambiguity, letters and reports are not edited. Depending on transfer agreements, referee reports obtained elsewhere may or may not be included in this compilation. Referee reports are anonymous unless the Referee chooses to sign their reports.)

23rd Sep 2021

Dear Dr. Lantero Rodríguez,

Thank you again for submitting your work to EMBO Molecular Medicine. We have now heard back from the three referees who evaluated your manuscript. As you will see from the reports below, the referees think that the study is interesting and acknowledge the quality and potential clinical relevance of the presented data. However, they raise a series of -primarily minor- concerns, which should be carefully addressed in a revision of the manuscript.

The referees' recommendations are rather clear, and there is no need to reiterate their comments. Importantly, referees raised issues regarding data accessibility. In line with the journal's data sharing policy, if practically possible and compatible with the individual consent agreement, we would ask you to deposit the data to a public database.

We would welcome the submission of a revised version within three months for further consideration. Please note that EMBO Molecular Medicine strongly supports a single round of revision. As acceptance or rejection of the manuscript will depend on another round of review, your responses should be as complete as possible.

EMBO Molecular Medicine has a "scooping protection" policy, whereby similar findings that are published by others during review or revision are not a criterion for rejection. Should you decide to submit a revised version, I do ask that you get in touch after three months if you have not completed it to update us on the status.

We are aware that many laboratories cannot function at full efficiency during the current COVID-19/SARS-CoV-2 pandemic and have therefore extended our "scooping protection policy" to cover the period required for a full revision to address the experimental issues. Please let me know should you need additional time, and also if you see a paper with related content published elsewhere.

Please read below for important editorial formatting and consult our author's guidelines for proper formatting of your revised article for EMBO Molecular Medicine.

I look forward to receiving your revised manuscript.

Use this link to login to the manuscript system and submit your revision: https://embomolmed.msubmit.net/cgi-bin/main.plex

Kind regards,
Jingyi

Jingyi Hou
Editor
EMBO Molecular Medicine

*****

When submitting your revised manuscript, please carefully review the instructions that follow below. We perform an initial quality control of all revised manuscripts before re-review; failure to include requested items will delay the evaluation of your revision.

We require:

1) A .docx formatted version of the manuscript text (including legends for main figures, EV figures and tables). Please make sure that the changes are highlighted to be clearly visible.

2) Individual production quality figure files as .eps, .tif, .jpg (one file per figure). For guidance, download the 'Figure Guide PDF': (https://www.embopress.org/page/journal/17574684/authorguide#figureformat).

3) A .docx formatted letter INCLUDING the reviewers' reports and your detailed point-by-point responses to their comments. As part of the EMBO Press transparent editorial process, the point-by-point response is part of the Review Process File (RPF), which will be published alongside your paper.

4) A complete author checklist, which you can download from our author guidelines

(https://www.embopress.org/page/journal/17574684/authorguide#submissionofrevisions). Please insert information in the checklist that is also reflected in the manuscript. The completed author checklist will also be part of the RPF.

5) Please note that all corresponding authors are required to supply an ORCID ID for their name upon submission of a revised manuscript.

6) It is mandatory to include a 'Data Availability' section after the Materials and Methods. Before submitting your revision, primary datasets produced in this study need to be deposited in an appropriate public database, and the accession numbers and database listed under 'Data Availability'. Please remember to provide a reviewer password if the datasets are not yet public (see https://www.embopress.org/page/journal/17574684/authorguide#dataavailability).

In case you have no data that requires deposition in a public database, please state so in this section. Note that the Data Availability Section is restricted to new primary data that are part of this study.

7) For data quantification: please specify the name of the statistical test used to generate error bars and P values, the number (n) of independent experiments (specify technical or biological replicates) underlying each data point and the test used to calculate p-values in each figure legend. The figure legends should contain a basic description of n, P and the test applied. Graphs must include a description of the bars and the error bars (s.d., s.e.m.). See also 'Figure Legend' guidelines: https://www.embopress.org/page/journal/17574684/authorguide#figureformat

8) We would also encourage you to include the source data for figure panels that show essential data. Numerical data should be provided as individual .xls or .csv files (including a tab describing the data). For blots or microscopy, uncropped images should be submitted (using a zip archive if multiple images need to be supplied for one panel). Additional information on source data and instruction on how to label the files are available at

.

9) Our journal encourages inclusion of *data citations in the reference list* to directly cite datasets that were re-used and obtained from public databases. Data citations in the article text are distinct from normal bibliographical citations and should directly link to the database records from which the data can be accessed. In the main text, data citations are formatted as follows: "Data ref: Smith et al, 2001" or "Data ref: NCBI Sequence Read Archive PRJNA342805, 2017". In the Reference list, data citations must be labeled with "[DATASET]". A data reference must provide the database name, accession number/identifiers and a resolvable link to the landing page from which the data can be accessed at the end of the reference. Further instructions are available at .

10) We replaced Supplementary Information with Expanded View (EV) Figures and Tables that are collapsible/expandable online. A maximum of 5 EV Figures can be typeset. EV Figures should be cited as 'Figure EV1, Figure EV2" etc... in the text and their respective legends should be included in the main text after the legends of regular figures.

- For the figures that you do NOT wish to display as Expanded View figures, they should be bundled together with their legends in a single PDF file called *Appendix*, which should start with a short Table of Content. Appendix figures should be referred to in the main text as: "Appendix Figure S1, Appendix Figure S2" etc.

- Additional Tables/Datasets should be labeled and referred to as Table EV1, Dataset EV1, etc. Legends have to be provided in a separate tab in case of .xls files. Alternatively, the legend can be supplied as a separate text file (README) and zipped together with the Table/Dataset file.

See detailed instructions here:

.

11) The paper explained: EMBO Molecular Medicine articles are accompanied by a summary of the articles to emphasize the major findings in the paper and their medical implications for the non-specialist reader. Please provide a draft summary of your article highlighting

- the medical issue you are addressing,

- the results obtained and

- their clinical impact.

This may be edited to ensure that readers understand the significance and context of the research. Please refer to any of our published articles for an example.

12) For more information: There is space at the end of each article to list relevant web links for further consultation by our readers. Could you identify some relevant ones and provide such information as well? Some examples are patient associations, relevant databases, OMIM/proteins/genes links, author's websites, etc...

13) Author contributions: the contribution of every author must be detailed in a separate section (before the acknowledgments).

14) A Conflict of Interest statement should be provided in the main text.

15) Every published paper now includes a 'Synopsis' to further enhance discoverability. Synopses are displayed on the journal webpage and are freely accessible to all readers. They include a short stand first (maximum of 300 characters, including space) as well as 2-5 one-sentences bullet points that summarizes the paper. Please write the bullet points to summarize the key NEW findings. They should be designed to be complementary to the abstract - i.e. not repeat the same text. We encourage inclusion of key acronyms and quantitative information (maximum of 30 words / bullet point). Please use the passive voice. Please attach these in a separate file or send them by email, we will incorporate them accordingly.

Please also suggest a striking image or visual abstract to illustrate your article as a PNG file 550 px wide x 300-600 px high.

EMBO Molecular Medicine has a "scooping protection" policy, whereby similar findings that are published by others during review or revision are not a criterion for rejection. Should you decide to submit a revised version, I do ask that you get in touch after three months if you have not completed it, to update us on the status.

***** Reviewer's comments *****

Referee #1 (Comments on Novelty/Model System for Author):

This study presents data indicating that p-tau 235 represents a late-preclinical (or relatively early in disease course) biomarker of AD pathology that could be useful for staging of disease.

Referee #1 (Remarks for Author):

This study by Lantero-Rodrigeuz, Snellman and coworkers describes a phosphorylation site in tau (aa 235) that temporally arises in the later stages of pre-clinical AD (i.e. A+T-) and is preceded by p-tau217 and 231. The authors identify p-tau235 in soluble brain homogenate from AD patients using IP-MS/MS. They subsequently measure p-tau235 in CSF in a discovery cohort of AD patients and controls and in two other cohorts (TRIAD and ALFA) to assess CSF levels at different stages of disease development. This a convincing and solid study and I have no comments for improvement in regard to the experiments.

Referee #2 (Comments on Novelty/Model System for Author):

The authors report a sequential development and characterization of a novel biomarker for staging preclinical Alzheimer's disease (P-tau235), starting exploratory work in brain tissue, characterizing positivity in sequential stages of the AD continuum in different clinical cohorts, and finally validate these findings against AD brains in different disease stages. a very well conducted study providing valuable insights into the pathophysiology of AD with possible clinical applicability.

Referee #2 (Remarks for Author):

Why do the authors not report blood measurements?

Minor:
Abbreviations in the text should be defined at first use. This is not the case now and highly impairs the flow of the text.

page 8, line 3: A+T needs to be: A+T-.

the clinical cohorts should be described briefly in the text, when they are referred to at first occurrence in the text. The cohorts are not known to all readers.

Referee #3 (Comments on Novelty/Model System for Author):

This reviewer has no concerns regarding the content of this manuscript

Referee #3 (Remarks for Author):

Under data availability the authors state that the data will not be deposited in an external repository. I would like to see a plan put in place that would allow for sharing of at least the measured concentrations from the two cohorts.

The above concern ties in with my only other comment on the paper - figure 6 is a log/log plot - while the authors do not try to make conclusions about the linearity of the data - without access to the raw data - it is hard to imagine what the plot would look like if the data was not log transformed before plotting.

Also, regarding the cut points used to define positivity for 231 and 235 levels: The text mentions that this is the mean of the normals + 2 standard deviations. Would it be possible to show these cutoffs on Figure 3/4a or maybe even generate a new figure to show the performance of this cutoff? I know they are shown in 6b - but those are log transformed data.

Institute of Neuroscience and Physiology
Department of Psychiatry and Neurochemistry
The Sahlgrenska Academy
University of Gothenburg

We want to thank the reviewers for their support and interesting suggestions. They shared some concerns, and a point-by-point response follows. The reviewer comments are quoted in bold, and our responses are in plain text. Additionally, all changes in the manuscript and appendix documents are now highlighted in yellow, making them easy to be located.

Referee #1

Referee #1 didn't refer any concerns.

We would like to thank referee #1 for the positive comments.

Referee #2

1. **"Why do the authors not report blood measurements?"**

We thank the referee for raising this important question. Our in-house SIMOA p-tau235 assay is at the moment only validated for measurements in CSF. P-tau levels in blood are approximately 10-100 fold lower than those in CSF, and at the time of the study, our assay lack the sensitivity necessary to successfully measure p-tau235 in all plasma samples. Our goal now is to further optimize our SIMOA p-tau235 so that we can overcome this sensitivity issue and reliably measure p-tau235 in all plasma samples.

2. **"Abbreviations in the text should be defined at first use. This is not the case now and highly impairs the flow of the text."**

We want to apologize for this clumsy mistake. This has now been properly corrected.

3. **"Page 8, line 3: A+T needs to be: A+T-."**

We want to thank the referee for spotting this errata. This has been now properly corrected.

Manuscript, Results, page 8: "*We found that CSF p-tau235 was mildly but significantly increased in the A+T- when compared with A-T- group (Cohen's d (d)$_{235}$ = 0.49, P <0.0001) (Fig. 4A), further supporting that this CSF biomarker increases in the preclinical stage of AD, when CSF Aβ$_{1-42/40}$ ratio is decreased but CSF p-tau181 is still not changed*."

4. **"The clinical cohorts should be described briefly in the text, when they are referred to at first occurrence in the text. The cohorts are not known to all readers."**

We understand referee's point and we concur. In the revised manuscript, a brief description of the clinical cohorts has been included in the results section, as this section precedes the methods section.

Manuscript, Results, page 6: "*The cohort comprised AD patients with typical AD CSF profile and neurological controls with minor neurological or psychiatric symptoms.*"

Manuscript, Results, page 7: "*The TRIAD cohort is a cross-sectional study with participants ranging across the AD continuum, with detailed clinical and cognitive assessment, and a wide range of biomarker data (both fluid and imaging).*"

Manuscript, Results, page 8: "*ALFA+ study is a cohort exclusively comprised by CU participants, and has the specific goal of investigating the preclinical stage of AD. Participants in ALFA+ are classified based on the presence (+/-) of Aβ (A) and tau pathology (T) in CSF into three groups: A-T-, A+T- and A+T+.*"

Referee #3

1. **Under data availability the authors state that the data will not be deposited in an external repository. I would like to see a plan put in place that would allow for sharing of at least the measured concentrations from the two cohorts.**

We understand the referee's point. We would like to express that all requests for raw and analysed data and materials will be promptly reviewed by the senior authors to verify whether the request is subject to any intellectual property or confidentiality obligations. Bulk anonymized data can be shared by request from any qualified investigator for the sole purpose of replicating procedures and results presented in the article, providing data transfer is in agreement with European Union legislation and decisions by the institutional review board of each participating center.

2. **The above concern ties in with my only other comment on the paper - figure 6 is a log/log plot - while the authors do not try to make conclusions about the linearity of the data - without access to the raw data - it is hard to imagine what the plot would look like if the data was not log transformed before plotting.**

Following the referee's suggestion, we have now changed Figure 6B and Appendix Figure 6B so that the correlation between CSF p-tau231 and p-tau235 now appears without log transformation. Figure text has been also modified to adapt the figure changes.

Figure 6B

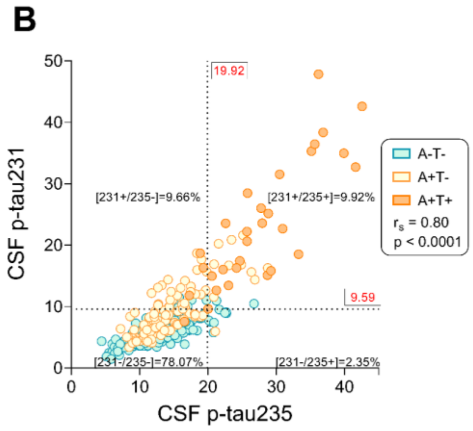

Manuscript, Figure legends, page 30: "*Correlation between CSF p-tau235 and p-tau231 in the whole ALFA+ cohort (Spearman's rank correlation: r_S = 0.80, P<0.0001).*"

Appendix Figure 6B

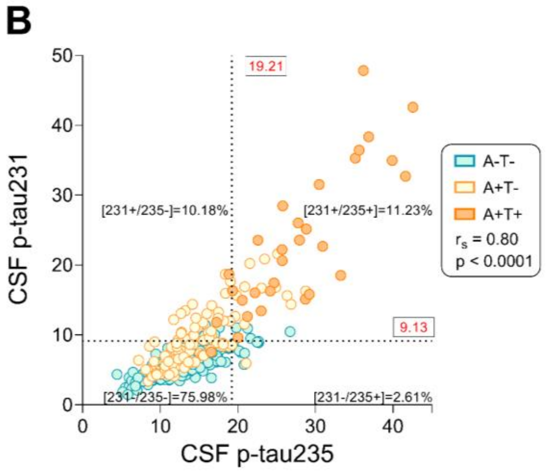

Appendix, Appendix Figure 6, page 11: *"Correlation between CSF p-tau235 and p-tau231 in the whole ALFA+ cohort (Spearman's rank correlation: r_S = 0.80, P<0.0001)."*

3. **Also, regarding the cut points used to define positivity for 231 and 235 levels: The text mentions that this is the mean of the normals + 2 standard deviations. Would it be possible to show these cutoffs on Figure 3/4a or maybe even generate a new figure to show the performance of this cutoff? I know they are shown in 6b - but those are log transformed data.**

Following the referee's suggestion, we have now changed Figure 3A and Figure 4A so that the cut-off value for CSF p-tau235 positivity is now visible for the reader. Figure text has been also modified to adapt the figure changes.

Figure 3A

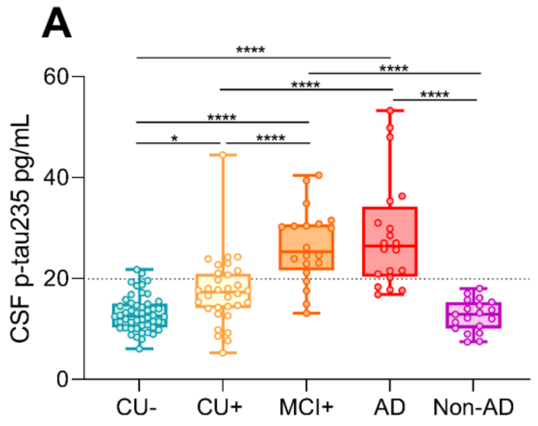

Manuscript, Figure legends, page 28: "*Cut-off value for CSF p-tau235 positivity is displayed with black dashed line (19.92 pg/mL)*" and "*CSF p-tau235 cut-off was determined as the mean + 2 SD of the A-T- group in ALFA+ cohort (19.92 pg/mL).*"

Figure 4A

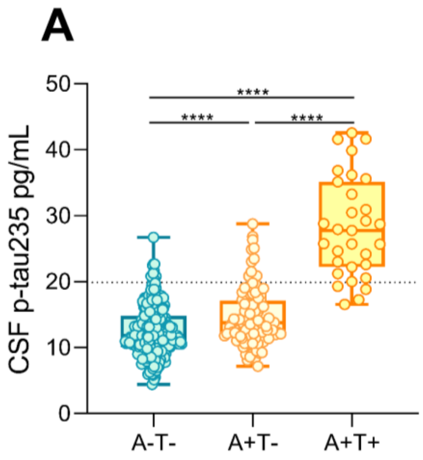

Manuscript, Figure legends, page 29: "*Cut-off value for CSF p-tau235 positivity is displayed with black dashed line (19.92 pg/mL)*" and "*CSF p-tau235 cut-off was determined as the mean + 2 SD of the A-T- group in ALFA+ cohort (19.92 pg/mL).*"

In connection with the previous point, we have now included the cut-offs values for CSF p-tau231 and p-tau235 positivity in Figure 6B and Appendix Figure 6B without log transformation, adapting the figure legends accordingly. Note that cut-offs in Figure 6A and Appendix Figure 6A are now clearly highlighted in red.

Manuscript, Figure legends, page 30: *"Assay cut-offs were determined as the mean + 2 SD of the A-T- group (defining p-tau231 and p-tau235 positivity or negativity in each participant). Cut-off values are indicated in red (19.92 and 9.59 pg/mL for CSF p-tau235 and p-tau231 respectively) and displayed with black dashed lines, resulting in four quadrants, each of them representing the four different positive or negatively status for each biomarker."*

Appendix, Appendix Figure 6, page 11: *"Assay cut-offs were determined as the 95th percentile of the A-T- group (defining p-tau231 and p-tau235 positivity or negativity in each participant). Cut-off values are indicated in red (19.21 and 9.13 pg/mL for CSF p-tau235 and p-tau231 respectively) and displayed with black dashed lines, resulting in four quadrants, each of them representing the four different positive or negatively status for each biomarker."*

Taken together, we have carried out the corrections as suggested and hope that you find our paper now acceptable for publication in EMBO Molecular Medicine. Many thanks for taking the time to review our manuscript and for kindly share with us your suggestions and remarks on how to improve our paper.

Sincerely,

Juan Lantero-Rodriguez and Kaj Blennow on behalf of all authors.

13th Oct 2021

Dear Dr. Lantero Rodríguez,

Thank you for the submission of your revised manuscript to EMBO Molecular Medicine. We have now received the enclosed report from the two referees who were asked to re-assess it. As you will see the referees are now overall supportive and I am pleased to inform you that we will be able to accept your manuscript pending the following amendments:

1. In the main manuscript file, please do the following:
- Remove the yellow color font.
- Multiple authors are missing from the Author contribution section. Please add all names.
- In Material and Methods, include a statement that the experiments conformed to the principles set out in the Human Services Belmont Report.

2. Appendix
- Appendix Table S5,6, & 7 are not called out. Please fix this.
- Please update all nomenclature (Add an S) to Appendix Table Sx / Figure Sx.

3. Data availability:
I noticed that in the cover letter you wrote "All requests for raw and analyzed data and materials will be promptly reviewed by the senior authors to verify whether the request is subject to any intellectual property or confidentiality obligations. Bulk anonymized data can be shared by request from any qualified investigator for the sole purpose of replicating procedures and results presented in the article, providing data transfer is in agreement with European Union legislation and decisions by the institutional review board of each participating center." I also understand that Referee #3 is not opposed to your answer regarding data sharing while he/she "would have liked to have anonymized data deposited externally".

Maybe I did not make it clear enough in my previous letter, but I wanted to point you to our author guidelines(https://www.embopress.org/page/journal/17574684/authorguide#datadeposition). According to the journal's data policy, if practically possible and compatible with the individual consent agreement, the authors should deposit the human clinical datasets to public databases at the time of publication. Usually, we recommend EGA (https://www.ebi.ac.uk/ega/home) for this purpose. Maybe you are not aware of this yet, but EGA does offer data-access control (https://ega-archive.org/access/data-access) through a data access committee which can be named by the authors.
Can you please let us know if you agree to deposit the anonymized data to a public database? Or is there any legal reason for not doing that?

4. Synopsis image: The text becomes somewhat blurry when the synopsis image is adjusted to the required resolution (550 px width, see attached). This can be solved by increasing the text size. Please provide a new image (PNG format, 550 px width x 400-600 px height) with better readability.

5. Synopsis text: I have slightly modified and shortened the synopsis text (see attached). Please let me know if it is fine as is or if you would like to introduce further modifications.

6. Our data editors have seen the manuscript, and they have made some comments and suggestions that need to be addressed (see attached). Please send back a revised version (in track change mode), as we will need to go through the changes.

7. The paper explained: I have made only minor modifications (see the same data-edited doc file).

8. We would also encourage you to include the source data for figure panels that show essential data. Numerical data should be provided as individual .xls or .csv files (including a tab describing the data). Additional information on source data and instruction on how to label the files are available at
.

9. As part of the EMBO Publications transparent editorial process initiative (see our Editorial at http://embomolmed.embopress.org/content/2/9/329), EMBO Molecular Medicine will publish online a Review Process File (RPF) to accompany accepted manuscripts.

In the event of acceptance, this file will be published in conjunction with your paper and will include the anonymous referee reports, your point-by-point response and all pertinent correspondence relating to the manuscript. Let us know whether you DO NOT agree with this and if you want to remove or keep any figures from it prior to publication.

Please note that the Authors checklist will be published at the end of the RPF.

I look forward to reading a new revised version of your manuscript as soon as possible.

Kind regards,
Jingyi

Jingyi Hou
Editor
EMBO Molecular Medicine

*** Instructions to submit your revised manuscript ***

*** PLEASE NOTE *** As part of the EMBO Publications transparent editorial process initiative (see our Editorial at https://www.embopress.org/doi/pdf/10.1002/emmm.201000094), EMBO Molecular Medicine will publish online a Review Process File to accompany accepted manuscripts.

In the event of acceptance, this file will be published in conjunction with your paper and will include the anonymous referee reports, your point-by-point response and all pertinent correspondence relating to the manuscript. If you do NOT want this file to be published, please inform the editorial office at contact@embomolmed.org.

To submit your manuscript, please follow this link:

https://embomolmed.msubmit.net/cgi-bin/main.plex

When submitting your revised manuscript, please include:

1) a .docx formatted version of the manuscript text (including Figure legends and tables)

2) Separate figure files*

3) supplemental information as Expanded View and/or Appendix. Please carefully check the authors guidelines for formatting Expanded view and Appendix figures and tables at https://www.embopress.org/page/journal/17574684/authorguide#expandedview

4) a letter INCLUDING the reviewer's reports and your detailed responses to their comments (as Word file).

5) The paper explained: EMBO Molecular Medicine articles are accompanied by a summary of the articles to emphasize the major findings in the paper and their medical implications for the non-specialist reader. Please provide a draft summary of your article highlighting
- the medical issue you are addressing,
- the results obtained and
- their clinical impact.
This may be edited to ensure that readers understand the significance and context of the research.
Please refer to any of our published articles for an example.

6) For more information: There is space at the end of each article to list relevant web links for further consultation by our readers. Could you identify some relevant ones and provide such information as well? Some examples are patient associations, relevant databases, OMIM/proteins/genes links, author's websites, etc...

7) Author contributions: the contribution of every author must be detailed in a separate section.

8) EMBO Molecular Medicine now requires a complete author checklist (https://www.embopress.org/page/journal/17574684/authorguide) to be submitted with all revised manuscripts. Please use the checklist as guideline for the sort of information we need WITHIN the manuscript. The checklist should only be filled with page numbers were the information can be found. This is particularly important for animal reporting, antibody dilutions (missing) and exact values and n that should be indicted instead of a range.

9) Every published paper now includes a 'Synopsis' to further enhance discoverability. Synopses are displayed on the journal webpage and are freely accessible to all readers. They include a short stand first (maximum of 300 characters, including space) as well as 2-5 one sentence bullet points that summarise the paper. Please write the bullet points to summarise the key NEW findings. They should be designed to be complementary to the abstract - i.e. not repeat the same text. We encourage inclusion of key acronyms and quantitative information (maximum of 30 words / bullet point). Please use the passive voice. Please attach these in a separate file or send them by email, we will incorporate them accordingly.

You are also welcome to suggest a striking image or visual abstract to illustrate your article. If you do please provide a jpeg file 550 px-wide x 400-px high.

10) A Conflict of Interest statement should be provided in the main text

11) Please note that we now mandate that all corresponding authors list an ORCID digital identifier. This takes <90 seconds to complete. We encourage all authors to supply an ORCID identifier, which will be linked to their name for unambiguous name identification.

Currently, our records indicate that the ORCID for your account is 0000-0002-7426-678X.

Please click the link below to modify this ORCID:
Link Not Available

12) The system will prompt you to fill in your funding and payment information. This will allow Wiley to send you a quote for the article processing charge (APC) in case of acceptance. This quote takes into account any reduction or fee waivers that you may be eligible for. Authors do not need to pay any fees before their manuscript is accepted and transferred to our publisher.

*Additional important information regarding Figures

Each figure should be given in a separate file and should have the following resolution:
Graphs 800-1,200 DPI
Photos 400-800 DPI
Colour (only CMYK) 300-400 DPI"

Figures are not edited by the production team. All lettering should be the same size and style; figure panels should be indicated by capital letters (A, B, C etc). Gridlines are not allowed except for log plots. Figures should be numbered in the order of their appearance in the text with Arabic numerals. Each Figure must have a separate legend and a caption is needed for each panel.

*Additional important information regarding figures and illustrations can be found at https://bit.ly/EMBOPressFigurePreparationGuideline. See also figure legend preparation guidelines: https://www.embopress.org/page/journal/17574684/authorguide#figureformat

The system will prompt you to fill in your funding and payment information. This will allow Wiley to send you a quote for the article processing charge (APC) in case of acceptance. This quote takes into account any reduction or fee waivers that you may be eligible for. Authors do not need to pay any fees before their manuscript is accepted and transferred to our publisher.

***** Reviewer's comments *****

Referee #2 (Comments on Novelty/Model System for Author):

the manuscript is well suited for publication

Referee #2 (Remarks for Author):

all issues have been resolved

Referee #3 (Comments on Novelty/Model System for Author):

I appreciate the updates to the manuscript and while I would have liked to have anonymized data deposited externally, I appreciate the legal issues with sharing patient data.

Referee #3 (Remarks for Author):

Thanks to the authors for the updates to the manuscript.

The authors have made all requested editorial changes.

18th Oct 2021

Dear Juan,

We are pleased to inform you that your manuscript is accepted for publication and is now being sent to our publisher to be included in the next available issue of EMBO Molecular Medicine.

We would like to remind you that as part of the EMBO Publications transparent editorial process initiative, EMBO Molecular Medicine will publish a Review Process File online to accompany accepted manuscripts. If you do NOT want the file to be published or would like to exclude figures, please immediately inform the editorial office via e-mail.

Please read below for additional IMPORTANT information regarding your article, its publication and the production process.

Thank you again for submitting this interesting work to EMBO Mol Med.

Jingyi

Jingyi Hou
Editor
EMBO Molecular Medicine

Follow us on Twitter @EmboMolMed
Sign up for eTOCs at embopress.org/alertsfeeds

*** *** *** IMPORTANT INFORMATION *** *** ***

SPEED OF PUBLICATION
The journal aims for rapid publication of papers, using using the advance online publication "Early View" to expedite the process: A properly copy-edited and formatted version will be published as "Early View" after the proofs have been corrected. Please help the Editors and publisher avoid delays by providing e-mail address(es), telephone and fax numbers at which author(s) can be contacted.

Should you be planning a Press Release on your article, please get in contact with embomolmed@wiley.com as early as possible, in order to coordinate publication and release dates.

LICENSE AND PAYMENT:

All articles published in EMBO Molecular Medicine are fully open access: immediately and freely available to read, download and share.

EMBO Molecular Medicine charges an article processing charge (APC) to cover the publication costs. You, as the corresponding author for this manuscript, should have already received a quote with the article processing fee separately. Please let us know in case this quote has not been received.

Once your article is at Wiley for editorial production you will receive an email from Wiley's Author Services system, which will ask you to log in and will present you with the publication license form for completion. Within the same system the publication fee can be paid by credit card, an invoice, pro forma invoice or purchase order can be requested.

Payment of the publication charge and the signed Open Access Agreement form must be received before the article can be published online.

PROOFS

You will receive the proofs by e-mail approximately 2 weeks after all relevant files have been sent o our Production Office. Please return them within 48 hours and if there should be any problems, please contact the production office at embopressproduction@wiley.com.

Please inform us if there is likely to be any difficulty in reaching you at the above address at that time. Failure to meet our deadlines may result in a delay of publication.

All further communications concerning your paper proofs should quote reference number EMM-2021-15098-V3 and be directed to the production office at embopressproduction@wiley.com.

Thank you,

Jingyi Hou
Editor
EMBO Molecular Medicine

EMBO PRESS

YOU MUST COMPLETE ALL CELLS WITH A PINK BACKGROUND ↓

PLEASE NOTE THAT THIS CHECKLIST WILL BE PUBLISHED ALONGSIDE YOUR PAPER

Corresponding Author Name: Juan Lantero-Rodriguez

Journal Submitted to: EMBO molecular medicine
Manuscript Number: EMM-2021-15098-V2

USEFUL LINKS FOR COMPLETING THIS FORM

http://www.antibodypedia.com
http://1degreebio.org
http://www.equator-network.org/reporting-guidelines/improving-bioscience-research-repor

http://grants.nih.gov/grants/olaw/olaw.htm
http://www.mrc.ac.uk/Ourresearch/Ethicsresearchguidance/Useofanimals/index.htm
http://ClinicalTrials.gov
http://www.consort-statement.org
http://www.consort-statement.org/checklists/view/32-consort/66-title
http://www.equator-network.org/reporting-guidelines/reporting-recommendations-for-tumo
http://datadryad.org
http://figshare.com
http://www.ncbi.nlm.nih.gov/gap
http://www.ebi.ac.uk/ega
http://biomodels.net/
http://biomodels.net/miriam/
http://jjj.biochem.sun.ac.za
https://osp.od.nih.gov/biosafety-biosecurity-and-emerging-biotechnology/
http://www.selectagents.gov/

Reporting Checklist For Life Sciences Articles (Rev. June 2017)

This checklist is used to ensure good reporting standards and to improve the reproducibility of published results. These guidelines are consistent with the Principles and Guidelines for Reporting Preclinical Research issued by the NIH in 2014. Please follow the journal's authorship guidelines in preparing your manuscript.

A- Figures
1. Data
The data shown in figures should satisfy the following conditions:

- the data were obtained and processed according to the field's best practice and are presented to reflect the results of the experiments in an accurate and unbiased manner.
- figure panels include only data points, measurements or observations that can be compared to each other in a scientifically meaningful way.
- graphs include clearly labeled error bars for independent experiments and sample sizes. Unless justified, error bars should not be shown for technical replicates.
- if n< 5, the individual data points from each experiment should be plotted and any statistical test employed should be justified
- Source Data should be included to report the data underlying graphs. Please follow the guidelines set out in the author ship guidelines on Data Presentation.

2. Captions

Each figure caption should contain the following information, for each panel where they are relevant:

- a specification of the experimental system investigated (eg cell line, species name).
- the assay(s) and method(s) used to carry out the reported observations and measurements
- an explicit mention of the biological and chemical entity(ies) that are being measured.
- an explicit mention of the biological and chemical entity(ies) that are altered/varied/perturbed in a controlled manner.

- the exact sample size (n) for each experimental group/condition, given as a number, not a range;
- a description of the sample collection allowing the reader to understand whether the samples represent technical or biological replicates (including how many animals, litters, cultures, etc.).
- a statement of how many times the experiment shown was independently replicated in the laboratory.
- definitions of statistical methods and measures:
  - common tests, such as t-test (please specify whether paired vs. unpaired), simple χ^2 tests, Wilcoxon and Mann-Whitney tests, can be unambiguously identified by name only, but more complex techniques should be described in the methods section;
  - are tests one-sided or two-sided?
  - are there adjustments for multiple comparisons?
  - exact statistical test results, e.g., P values = x but not P values < x;
  - definition of 'center values' as median or average;
  - definition of error bars as s.d. or s.e.m.

Any descriptions too long for the figure legend should be included in the methods section and/or with the source data.

In the pink boxes below, please ensure that the answers to the following questions are reported in the manuscript itself. Every question should be answered. If the question is not relevant to your research, please write NA (non applicable). We encourage you to include a specific subsection in the methods section for statistics, reagents, animal models and human subjects.

B- Statistics and general methods

Please fill out these boxes ↓ (Do not worry if you cannot see all your text once you press return)

1.a. How was the sample size chosen to ensure adequate power to detect a pre-specified effect size?	NA
1.b. For animal studies, include a statement about sample size estimate even if no statistical methods were used.	NA
2. Describe inclusion/exclusion criteria if samples or animals were excluded from the analysis. Were the criteria pre-established?	Inclusion and exclusion criteria is fully described in methods section
3. Were any steps taken to minimize the effects of subjective bias when allocating animals/samples to treatment (e.g. randomization procedure)? If yes, please describe.	NA
For animal studies, include a statement about randomization even if no randomization was used.	NA
4.a. Were any steps taken to minimize the effects of subjective bias during group allocation or/and when assessing results (e.g. blinding of the investigator)? If yes please describe.	Brain measurements and biomarker measurements were performed blinded
4.b. For animal studies, include a statement about blinding even if no blinding was done	NA
5. For every figure, are statistical tests justified as appropriate?	All figures include the statistical methods perfomed
Do the data meet the assumptions of the tests (e.g., normal distribution)? Describe any methods used to assess it.	Normality of the distribution for each biomarker was tested using the Kolmogorov-Smirnov test combined with visual inspection of histograms.
Is there an estimate of variation within each group of data?	Homoscedasticity was tested
Is the variance similar between the groups that are being statistically compared?	The assumptions of the tests were all met

C- Reagents

6. To show that antibodies were profiled for use in the system under study (assay and species), provide a citation, catalog number and/or clone number, supplementary information or reference to an antibody validation profile. e.g., Antibodypedia (see link list at top right), 1DegreeBio (see link list at top right).	All reagents used in the study ae described in the methods section and suppliers are included
7. Identify the source of cell lines and report if they were recently authenticated (e.g., by STR profiling) and tested for mycoplasma contamination.	NA

* for all hyperlinks, please see the table at the top right of the document

D- Animal Models

8. Report species, strain, gender, age of animals and genetic modification status where applicable. Please detail housing and husbandry conditions and the source of animals.	NA
9. For experiments involving live vertebrates, include a statement of compliance with ethical regulations and identify the committee(s) approving the experiments.	NA
10. We recommend consulting the ARRIVE guidelines (see link list at top right) (PLoS Biol. 8(6), e1000412, 2010) to ensure that other relevant aspects of animal studies are adequately reported. See author guidelines, under 'Reporting Guidelines'. See also: NIH (see link list at top right) and MRC (see link list at top right) recommendations. Please confirm compliance.	NA

E- Human Subjects

11. Identify the committee(s) approving the study protocol.	Ethical approval for human brain cohort was granted by London Central Research Ethics Committee. Ethical approval for research use of discovery cohort samples has been granted by the Ethics Committee at the University of Gothenburg, Sweden. The TRIAD study was approved by the Research Ethics Board of the Montreal Neurological Institute and the Faculty of Medicine Research Ethics Office (McGill University, Canada). The ALFA+ study was approved by the Independent Ethics Committee "Parc de Salut Mar", Barcelona, Spain.
12. Include a statement confirming that informed consent was obtained from all subjects and that the experiments conformed to the principles set out in the WMA Declaration of Helsinki and the Department of Health and Human Services Belmont Report.	All participants provided written consent and all experiments conformed to the principles set out in the WMA Declaration of Helsinki and Human Services Belmont Report
13. For publication of patient photos, include a statement confirming that consent to publish was obtained.	NA
14. Report any restrictions on the availability (and/or on the use) of human data or samples.	No restrictions
15. Report the clinical trial registration number (at ClinicalTrials.gov or equivalent), where applicable.	NA
16. For phase II and III randomized controlled trials, please refer to the CONSORT flow diagram (see link list at top right) and submit the CONSORT checklist (see link list at top right) with your submission. See author guidelines, under 'Reporting Guidelines'. Please confirm you have submitted this list.	NA
17. For tumor marker prognostic studies, we recommend that you follow the REMARK reporting guidelines (see link list at top right). See author guidelines, under 'Reporting Guidelines'. Please confirm you have followed these guidelines.	NA

F- Data Accessibility

18: Provide a "Data Availability" section at the end of the Materials & Methods, listing the accession codes for data generated in this study and deposited in a public database (e.g. RNA-Seq data: Gene Expression Omnibus GSE39462, Proteomics data: PRIDE PXD000208 etc.) Please refer to our author guidelines for 'Data Deposition'.<br><br>Data deposition in a public repository is mandatory for:<br>a. Protein, DNA and RNA sequences<br>b. Macromolecular structures<br>c. Crystallographic data for small molecules<br>d. Functional genomics data<br>e. Proteomics and molecular interactions	NA
19. Deposition is strongly recommended for any datasets that are central and integral to the study; please consider the journal's data policy. If no structured public repository exists for a given data type, we encourage the provision of datasets in the manuscript as a Supplementary Document (see author guidelines under 'Expanded View' or in unstructured repositories such as Dryad (see link list at top right) or Figshare (see link list at top right).	All requests for raw and analyzed data and materials will be promptly reviewed by the senior authors to verify whether the request is subject to any intellectual property or confidentiality obligations. Bulk anonymized data can be shared by request from any qualified investigator for the sole purpose of replicating procedures and results presented in the article, providing data transfer is in agreement with European Union legislation and decisions by the institutional review board of each participating center.
20. Access to human clinical and genomic datasets should be provided with as few restrictions as possible while respecting ethical obligations to the patients and relevant medical and legal issues. If practically possible and compatible with the individual consent agreement used in the study, such data should be deposited in one of the major public access-controlled repositories such as dbGAP (see link list at top right) or EGA (see link list at top right).	NA
21. Computational models that are central and integral to a study should be shared without restrictions and provided in a machine-readable form. The relevant accession numbers or links should be provided. When possible, standardized format (SBML, CellML) should be used instead of scripts (e.g. MATLAB). Authors are strongly encouraged to follow the MIRIAM guidelines (see link list at top right) and deposit their model in a public database such as Biomodels (see link list at top right) or JWS Online (see link list at top right). If computer source code is provided with the paper, it should be deposited in a public repository or included in supplementary information.	NA

G- Dual use research of concern

22. Could your study fall under dual use research restrictions? Please check biosecurity documents (see link list at top right) and list of select agents and toxins (APHIS/CDC) (see link list at top right). According to our biosecurity guidelines, provide a statement only if it could.	NA
